# Mechanistic Insights into the Triplet Sensitized Photochromism of Diarylethenes

**DOI:** 10.1002/chem.202000877

**Published:** 2020-05-26

**Authors:** Sebastian Fredrich, Tobias Morack, Michel Sliwa, Stefan Hecht

**Affiliations:** ^1^ Department of Chemistry & IRIS Adlershof Humboldt-Universität zu Berlin Brook-Taylor-Strasse 2 12489 Berlin Germany; ^2^ Univ. Lille, CNRS, UMR 8516 – LASIR – Laboratoire de, Spectrochimie Infrarouge et Raman F-59000 Lille France; ^3^ DWI—Leibniz Institute for Interactive Materials Forckenbeckstr. 50 52074 Aachen Germany; ^4^ Institute of Technical and Macromolecular Chemistry RWTH Aachen University Worringer Weg 2 52074 Aachen Germany

**Keywords:** diarylethenes, fatigue resistance, photochromism, transient absorption, triplet

## Abstract

Operating photoswitchable molecules repetitively and reliably is crucial for most of their applications, in particular in (opto)electronic devices, and related to reversibility and fatigue resistance, which both critically depend on the photoisomerization mechanism defined by the substitution pattern. Two diarylethene photoswitches bearing biacetyl triplet sensitizers either at the periphery or at the core were investigated using both stationary as well as transient UV/Vis absorption spectroscopy ranging from the femtosecond to the microsecond time scale. The diarylethene with two biacetyl moieties at the periphery is switching predominantly from the triplet excited state, giving rise to an enhanced fatigue resistance. In contrast, the diarylethene bearing one diketone at the photoreactive inner carbon atom cyclizes from the singlet excited state and shows significantly higher quantum yields for both cyclization and cycloreversion.

## Graphical abstract

**Photoswitching of diarylethenes** from the triplet excited state is highly dependent on the substitution pattern defining the location of the internal triplet sensitizer. Fatigue occurs predominantly via a singlet pathway whereas electronic decoupling of the sensitizer increases photoisomerization quantum yields in both directions.

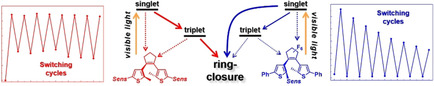

## Introduction

In order to be incorporated into devices, photoactive materials need to exhibit a precise and typically repeatable response to (localized) light stimuli. In this context, photoswitches have been heavily explored over the past decades and among them diarylethenes (DAEs)^1^ have proven exceptionally useful due to their thermal bistability and ability to modulate fluorescence^2^ and electronic properties.^3^ This has led to the development of optical transistors^4^ as well as storage media^5^ based on optical writing and erasing of DAE‐based materials. Although this class of photochromic compounds has been structurally optimized to improve its performance, in particular with regard to excitation wavelengths,^6^ quantum yields,^7^ and HOMO–LUMO modulation,^8^ fatigue due to photochemical byproduct formation^9^ remains an issue, in solution but also in the solid state. To prevent UV‐induced byproduct formation, several approaches have been followed, for example by sterically blocking and suppressing radical reactivity^10^ or by the incorporation of electron‐withdrawing moieties.4b Clearly, in order to develop an extraordinarily robust DAE, it is essential to fully understand the mechanism of the desired ring closure and opening as well as the competing photoreactions. Several theoretical^11^ as well as time‐resolved spectroscopy studies^12^ have been carried out to determine the involved excited states and their role in the switching process for classical DAEs. Depending on the actual structure, switching has been reported to occur primarily from a hot vibrationally singlet excited state (Franck–Condon (FC) state) as well as from the relaxed singlet state but also from triplet and higher excited states.^13^

**Scheme 1:**
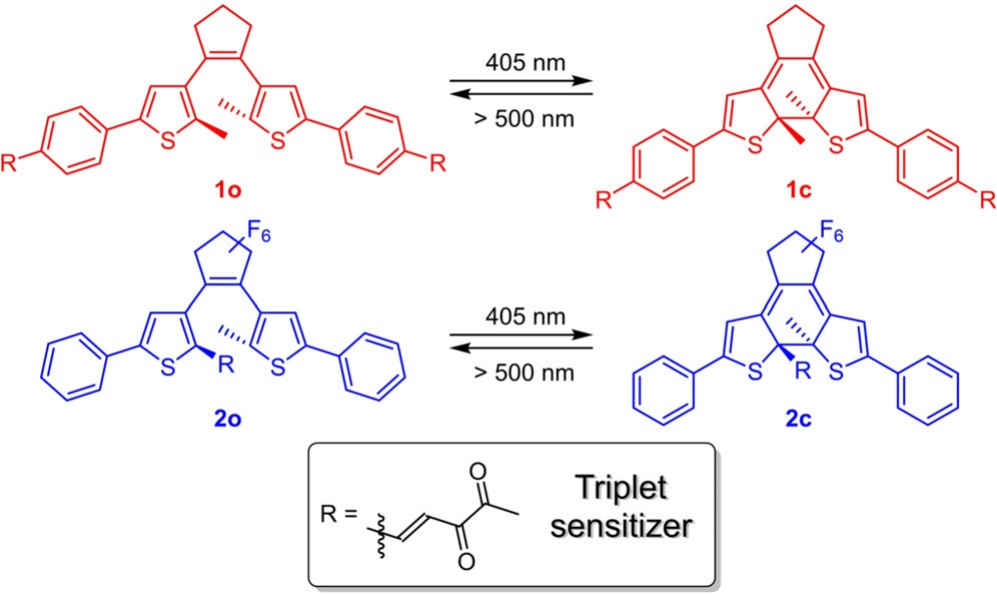
Investigated diarylethenes bearing a biacetyl sensitizer at the periphery (**1**, red)6b or in the central position (**2**, blue).

We have recently demonstrated that combining a triplet pathway with visible light excitation results in the remarkably stable DAE **1** (Scheme 1).6b Our approach took advantage of introducing two biacetyl moieties at the periphery of the DAE via a π‐conjugated vinylene bridge to lead to efficient population of the triplet excited state upon blue‐light excitation (405 nm) of the bathochromically shifted diketone absorption band (*λ*_max_=390 nm) with a much increased molar extinction coefficient (*ϵ*(390 nm)=27,000 m ^−1^ cm^−1^) as compared to parent biacetyl. As a result, DAE **1** underwent efficient ring closure upon visible light excitation (in the absence of oxygen, which acts as triplet quencher) and displayed unique long‐term photostability, which we have been ascribing to the fact that switching primarily occurs on the triplet manifold.6b The enhanced fatigue resistance of DAE **1** was, however, accompanied by reduced quantum yields of ring opening, which requires population of the reactive singlet excited state.^14^

In this article, we report on the excited state dynamics of two DAE photoswitches carrying triplet sensitizing moieties. In addition to DAE **1**, we are describing DAE **2** as an alternative structure, in which one biacetyl unit is introduced directly at the central hexatriene system via a vinylene spacer to assure for π‐conjugation in the open isomer yet not in the closed isomer. In the latter, we are expecting a less efficient intersystem crossing (ISC) and thus improved ring opening quantum yield as compared to DAE **1**. We are discussing the isomerization pathways of both DAEs, which have been investigated via transient absorption spectroscopy to reveal the involved excited states and compare their reactivities, in particular with regard to the point of attaching the sensitizing moiety.

## Results and Discussion

The “ideal” DAE should combine several properties to exhibit optimal switching behavior: (i) The absorption of the open isomer should be shifted from the UV to the visible range to utilize excitation with lower energies to affect ring closure; (ii) ring closure should result from the triplet excited state to avoid potential side reactions originating from the singlet excited state; (iii) the absorption of the closed isomer should be in the red region of the visible spectrum to allow for its selective excitation and ring opening at low excitation energies; and (iv) ring opening should occur with reasonable cycloreversion quantum yield, which requires sufficient population of the singlet excited state and thus limited ISC. DAE **2** was designed to fulfill these requirements by attaching the triplet sensitizing diketone unit to the inner 2‐position of one of the thiophene moieties (Scheme 1). On the one hand, the absorption of the open isomer **2 o** should be extended towards the visible range because of the increased length of the π‐system similarly to DAE **1**. More importantly, electronic excitation of **2 o**, in which π‐conjugation between the DAE core and the sensitizing diketone unit is assured, should lead to efficient ISC and predominant population of the triplet excited state. On the other hand, upon ring closure to the closed isomer **2 c** the diketone sensitizer is decoupled from the DAE π‐system since the resulting sp^3^‐hybridized thiophene C‐2 atom impedes electronic communication. Consequently, low energy, visible light excitation of the DAE should lead to increased population of the singlet excited state due to less efficient ISC and thus enhanced ring opening when compared to **1 c**. In addition, the absorption of **2 c** is expected to be hypsochromically shifted when compared to **1 c** and it is also expected to display hypochromic behavior due to the shorter π‐system and non‐symmetrical substitution pattern. A DAE bearing diketone motifs at both inner 2‐positions of the two thiophene moieties in a symmetric fashion was not considered due to anticipated steric hindrance during ring closure.

**Figure 1:**
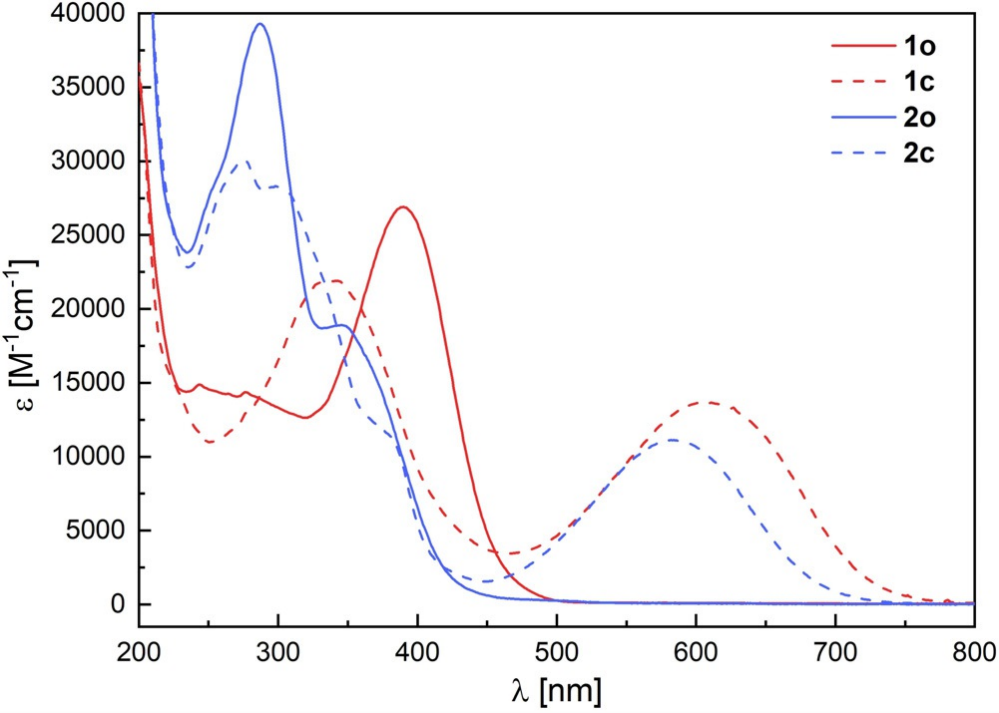
Absorption spectra of investigated DAE derivatives as both isomers: **1** (red) and **2** (blue) in their open (solid) and closed (dashed) forms (molar extinction coefficient derived from solutions in CH_3_CN, 4×10^−5^ m, 25 °C).

An acetonitrile solution of **2** shows the characteristic absorption pattern of the open form of a non‐symmetrically substituted DAE derivative (Figure 1). The intense absorbance band at *λ*_max_=289 nm corresponds to the overlap of the absorption of both electronically decoupled aryl moieties. The shoulder at *λ*_max_=357 nm is attributed to the half of the DAE with longer π‐conjugated system bearing the diketone moiety. This lowest energy transition of **2 o** is slightly blue‐shifted and less intense when compared to **1 o**, which can be explained by the interaction between the thiophene donor and diketone acceptor units that extends over a longer π‐system in **1 o** including the intermediate phenyl moiety.

Upon irradiation with blue light (*λ*_irr_=405 nm), the build‐up of a red‐shifted broad band with a maximum in the visible range (*λ*_max_=582 nm) can be observed. This can be assigned to the formation of the closed isomer and is also accompanied by a change in the spectral signature in the UV‐region showing new maxima at 273, 314, and 384 nm. As expected, the long wavelength absorbance of **2 c** is blue‐shifted when compared to the one of **1 c**, yet it is essentially separated from the absorption of **2 o**.

Conversion to **2 c** at 405 nm is quantitative (see Table 1), a result that was also observed for **1 o**→**1 c** and that is important for further applications of this molecule. The quantum yield of the ring closure (*Φ*_o→c_) was determined under degassed conditions since oxygen can act as triplet state quencher.^15^ This process is not only lowering the efficiency (and therefore the quantum yield) of the cyclization, but the thus‐generated singlet oxygen can irreversibly oxidize the photoswitch, leading to considerable fatigue. A quantum yield of *Φ*_o→c_=0.55 was determined for the ring closure, which is significantly higher than for **1** and also slightly exceeds the typically considered maximum of 0.5 for common DAEs, which is based on an equal distribution of the photoreactive antiparallel and unreactive parallel conformation, in addition to an efficient ultrafast cyclization from singlet excited state.^16^ In the case of **2 o**, the bulky internal vinylene diketone substituent presumably destabilizes the parallel form, thus leading to a larger fraction of the antiparallel conformer in solution.^17^ In addition, there appears to be a significant rotational barrier between both conformers as indicated by two distinct signals observed in the room temperature ^1^H NMR of **2 o** with a relative integral ratio of 52:48 proving the unequal distribution of both conformers (see Figure S21 in the Supporting Information). In view of the different solvents (CH_3_CN for quantum yield determination vs. CDCl_3_ for NMR spectroscopy) as well as measurement errors, the quantum yield and conformational equilibrium values are in reasonable agreement.

**Table 1:** Summary of stationary photochemical data of the investigated diarylethenes (4×10^−5^ m, CH_3_CN, 25 °C).

DAE	*λ*_irr_ [nm]	*Φ* [at *λ*_irr_] degassed	*Φ* [at *λ*_irr_] non‐degassed	PSS [at *λ*_irr_] (open:closed)
**1** 6b	313 (o→c) 405 (o→c) 577 (c→o)	0.18 0.30 0.0003	0.005 0.009 0.0003	34:66 0:100 100:0
**2**	289 (o→c) 365 (o→c) 405 (o→c) 577 (c→o)	0.49 0.55 0.55 0.063	0.54 0.51 0.41 0.060	0:100 0:100 0:100 100:0

**Figure 2:**
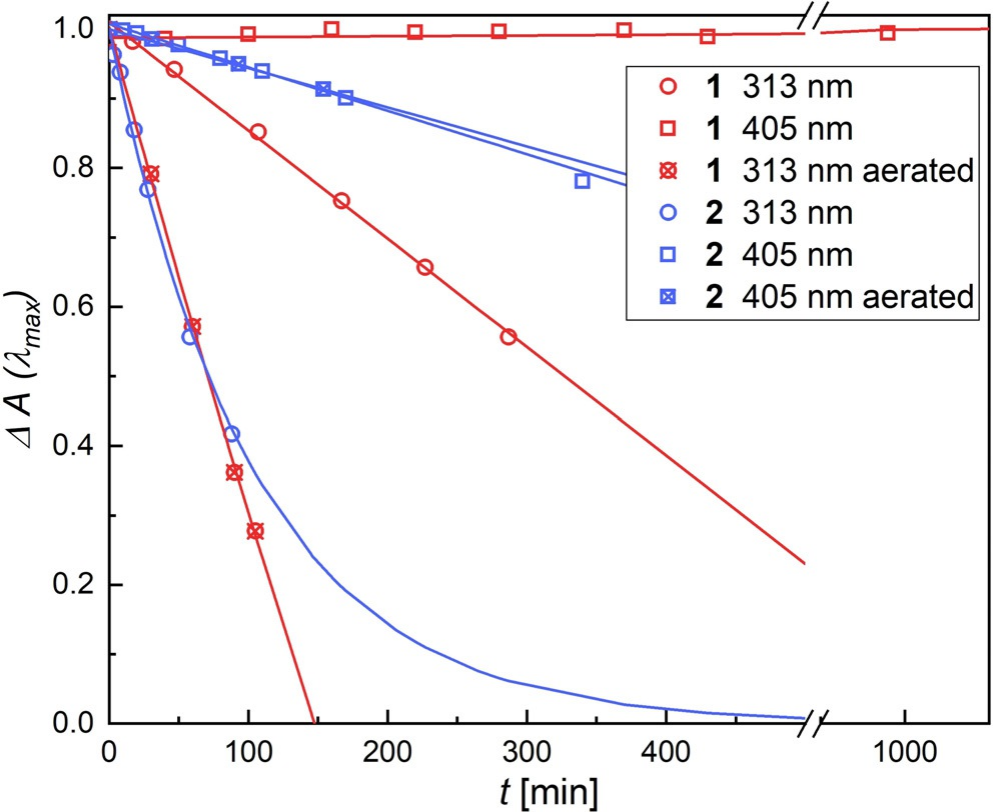
Long‐term irradiation of degassed (open symbols) or non‐degassed (crossed symbols) CH_3_CN‐solutions of **1** (red), **2** (blue, each 4×10^−5^ m, 25 °C) with a 1000 W continuum lamp using bandpass‐filters (313 nm: circles, 405 nm: squares). The decay of normalized absorption at the band maximum of the closed isomer (**1**: 608 nm, **2**: 582 nm) illustrates the diminishing concentration of diarylethene and increasing amount of byproduct. The lines serve as guide to the eye only.

Ring closure of **1 o** via the triplet excited state was initially proven by quenching experiments, of which the easiest one is the measurement of the quantum yield under non‐degassed conditions. In an aerated solution, oxygen is present in a much higher concentration than the investigated DAE (ca. 2×10^−3^ m as compared to 4×10^−5^ m in acetonitrile)^18^ and able to quench triplet excited states and photochemical pathways originating from them. Surprisingly, the ring closure quantum yield at 405 nm of **2 o** in non‐degassed acetonitrile was determined to be still quite high with *Φ*=0.41. This contradicts our initial hypothesis of a solely operating triplet cyclization pathway and could result in significant fatigue upon irradiation. To investigate this, **2 o** was exposed to continuous irradiation with light of *λ*_irr_=405 nm and the development of the absorbance of the closed isomer was monitored (Figure 2) as indication of side reactions, usually bleaching the compound and forming a hypsochromically absorbing byproduct.

Comparing the fatigue behavior of **1** and **2**, it becomes clear that **1** is not showing any fatigue over several hours of intense irradiation, whereas **2** shows a considerable decrease of the band of the closed isomer during the same duration and under identical conditions. As expected from the destructive effect of high‐energy irradiation, the fatigue behavior of **1** and **2** is highly wavelength‐dependent, showing significant decomposition upon exposure to UV‐light with a wavelength of 313 nm.9a The stability of **1** decreases as well under atmospheric conditions, that is, without flushing the sample with argon, and oxidative side‐reactions originating from singlet oxygen are supposed to be responsible for this degradation. However, such a dependence on the presence of oxygen was not found for DAE **2**.

These results indicate that ring closure does not exclusively occur from the triplet state for **2 o** in contrast to **1 o**. To further investigate this behavior, transient absorption spectroscopy was used to get insight into the excitation pathways. Whereas diarylethene cyclization from the singlet state is known to be very fast, occurring in the hundreds of femtoseconds range,12c, 12d triplet excited states are typically long‐lived in the microsecond time window. For this reason, first the behavior of **1** and **2** was investigated using nanosecond flash photolysis, that is, nano‐ to microsecond UV/Vis transient absorption spectroscopy.

The exact set‐up has been detailed elsewhere,^19^ but in this work for instance: An initial nanosecond laser pulse of 1 mJ with a wavelength of *λ*=355 nm was applied to samples of the DAEs **1** or **2**, respectively, dissolved in acetonitrile with a concentration adjusted to an absorbance of *A*=1 at irradiation wavelength (5–7×10^−5^ m). The absorbance difference (with laser minus without laser) evolution for different wavelengths was monitored. The initial pulse had a width of 8 nanoseconds, and only triplet excited state and photo‐product evolution are observed in this time scale.

**Figure 3:**
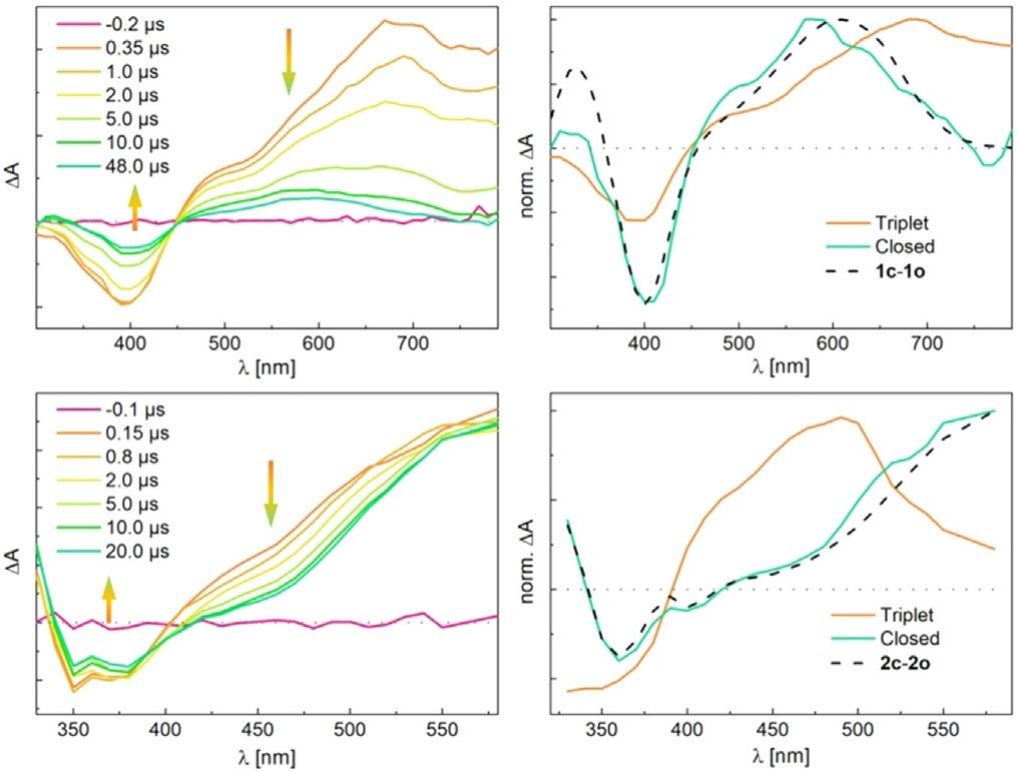
Transient absorption difference spectra upon nanosecond laser pulse excitation (*λ*_max_=355 nm) for selected times under degassed conditions of **1 o** (top left) and **2 o** (bottom left); evolution associated difference spectra of the triplet excited states and closed forms **1 c** (top right) and **2 c** (bottom right) obtained from a sequential model as well as the difference spectra of closed and open forms obtained from stationary experiments (dashed lines). Zero‐absorbance lines are given as dotted lines.

A solution of **1 o** was prepared under degassed conditions by flushing with argon and was measured accordingly. The initial formation of a broad positive transient band at around *λ*_max_=680 nm could be observed as well as a negative absorption that can be ascribed to the bleaching of the stationary band of the open isomer at around 390 nm (Figure 3, top left). Both bands decay with a time constant of *τ*=2.9 μs that is typical for the triplet excited state of the parallel conformer (see also Figure S4 in the Supporting Information).12a In accordance with the stationary experiments, after 50 μs the initial spectrum is not fully recovered, but a new band appears at around 580 nm corresponding to the formation of the closed isomer **1 c**. Only one time constant is found for the decay of the antiparallel and parallel triplet excited state. Assuming an equal quantum yield for the formation of the ISC for both conformers, the depopulation band recovery is characteristic of relaxation to the open form (without cyclization) from the parallel triplet excited state, cyclization and relaxation to the open form from the antiparallel triplet excited state. About 30 % of the depopulation remains that is in agreement with the cyclization quantum yield of compound **1** (Table 1). Noteworthy, the transient profile can be fitted accurately by only one decay time constant and no deviating behavior of the parallel and antiparallel conformers was observed.

The spectrum of the closed isomer obtained from the transient data of **1** can be reproduced well by the difference spectrum of **1 c** and **1 o** from the stationary experiment, verifying the correct fitting (Figure 3, top right).

A second sample of **1 o** was now measured under air in non‐degassed conditions. The presence of oxygen as quencher should have a significant effect on the lifetime of the triplet excited state and indeed, the spectral pattern obtained for the non‐degassed solution is decaying with *τ*=200 ns and thus much faster than under argon (see Figure S3 in the Supporting Information). The complete recovery of the depopulation band as well as the absence of a newly formed band at the end of the recorded time (5 μs) are in agreement with the low quantum yield under non‐degassed conditions.

Samples of **2 o** were prepared analogously as for **1 o** and measured under degassed conditions. Upon excitation with *λ*=355 nm, a transient absorption spectrum with a depopulation band and a positive band at 580 nm was observed (Figure 3, bottom left). The positive band has the same maximum as the absorption spectrum of the closed form but has a shoulder at 480 nm. The shoulder feature and depopulation decay with a time constant of *τ*=3.8 μs and is assigned to triplet relaxation of the parallel form.^12^ After its decay, the difference absorption spectrum of the closed form remains. It should be stressed here that the absorbance at 580 nm is constant and in agreement with the quantum yield measurements. These results suggest ring closure to occur directly from the singlet excited state in case of **2 o**. Furthermore, upon irradiation under non‐degassed conditions, the spectral features of **2 c** appear immediately with the initial pulse while no intermediate long‐lived transient absorption band indicating the presence of a triplet excited state could be observed (compare Figure S14 in the Supporting Information). Similar to the literature, the triplet transient band observed in the degassed sample could be ascribed mainly to the parallel conformation in solution, which is not able to undergo ring closure.

Formation of the triplet excited state and closed form of **2** occur on the sub nanosecond time scale. Femtosecond transient absorption experiments were thus conducted to have a more detailed insight into their formation.

**Figure 4:**
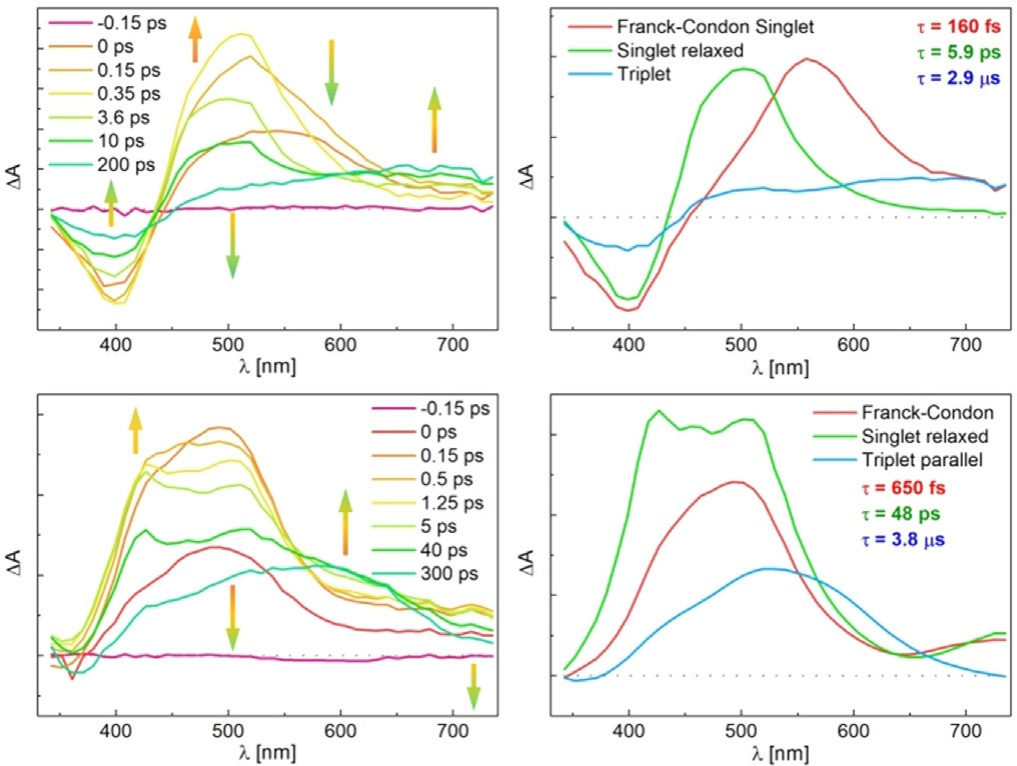
Transient difference spectra of upon 100 femtosecond laser pulse excitation (*λ*_max_=320 nm) for selected times under degassed conditions of **1 o** (top left) and **2 o** (bottom left); evolution associated spectra of **1 o** (top right) and **2 o** (bottom right) obtained from a sequential model. Zero‐absorbance lines are given as dotted lines.

**Scheme 2:**
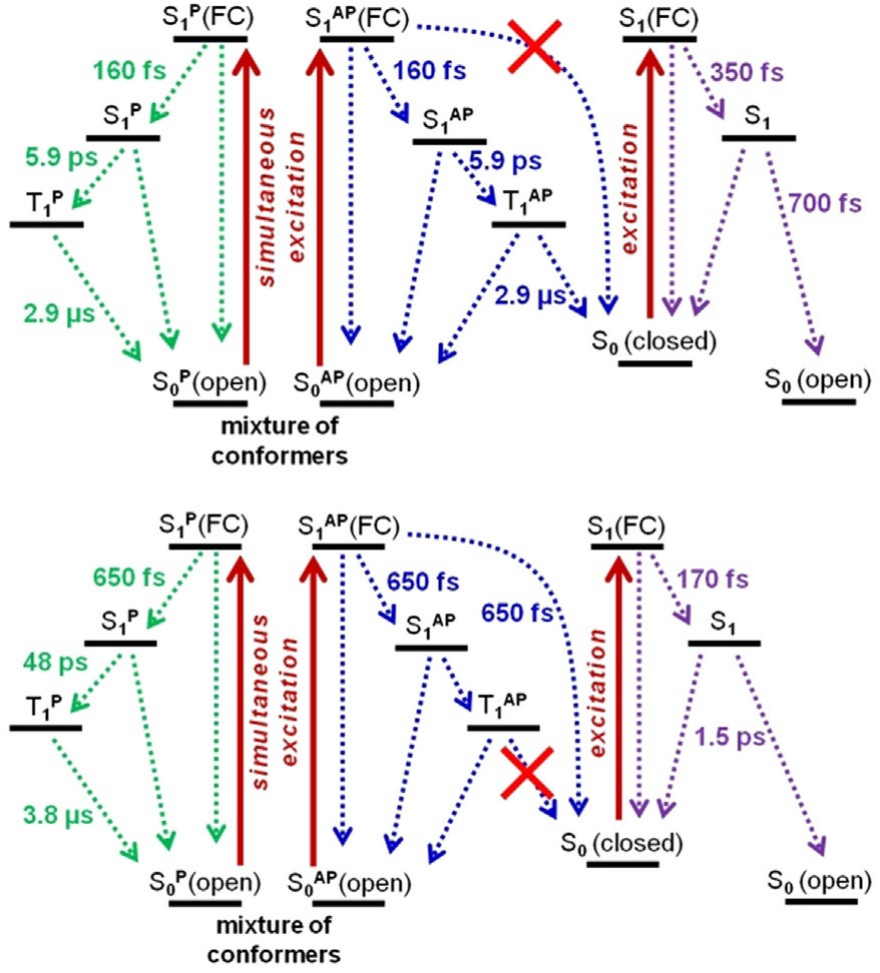
Relaxation schemes after the photoexcitation of: **1 o** with 400 nm and 355 nm as well as for **1 c** with 640 nm (top); **2 o** with 400 nm and 355 nm as well as for **2 c** with 640 nm and 355 nm (bottom).

The data obtained for a degassed sample of **1 o** excited with a 150 fs pulse at 320 nm are summarized in Figure 4 (top left). The initial transient absorption spectrum at 0 ps is characterized by a broad positive band with a maximum at around 560 nm assigned to the FC state (a hot, vibrationally singlet excited state) and the depopulation band. It is relaxing in 160 fs to the cold singlet excited state comprising a signature with a maximum at 500 nm. This vibrational relaxation is much faster than the subsequent ISC with a characteristic time of 5.9 ps towards the triplet state with a broad maximum at around 670 nm and the presence of an isosbestic point at 590 nm, signature of a sequential mechanism. A rather short time constant like this was observed before for organic triplet sensitizers such as benzophenone.^20^ The data can be accurately fitted by sequential dynamics. It means that the parallel and antiparallel forms have quite similar decay times and spectral separation is not possible. The picoseconds time constant for the decay of the singlet excited state is in agreement with the absence of cyclization from this state. (Scheme 2, top).

In contrast, compound **2 o** shows distinct decay times for its conformers during the relaxation process in the femtosecond range (Figure 4). First, the two FC states evolve simultaneously with the initial pulse to a superposition band located roughly at 500 nm. The transient absorption spectrum showed a broad positive absorption band that is growing in about 650 femtoseconds with three maxima at 430, 460 and 500 nm with a tail >600 nm (0.5 ps spectrum). This is characteristic for the formation of both S_1_^AP^ (antiparallel) and S_1_^P^ (parallel) relaxed states from FC states. At the same time, the closed isomer is formed from the antiparallel Franck–Condon state. The band at 460 nm disappears and is thus assigned to the decay of S_1_^AP^ towards the ground state. Then, in few tens of picoseconds, the bands at 430 and 500 nm decay and a broad transient absorption remains equal to the first spectra measured at 100 ns in the other setup. Therefore, the bands located at 430 nm and 500 nm are assigned to the S_1_^P^ state. The singlet excited state of the parallel conformer is slowly undergoing ISC to the triplet excited state with a time constant of *τ*=48 ps. These findings are in agreement with the results obtained from the nanosecond measurements and confirm that ring closure of **2 o**, in contrast to **1 o**, occurs via the singlet excited state.

The ring opening event was also analyzed as well using femtosecond to millisecond transient absorption spectroscopies. As expected, no reactive triplet excited state was found in case of **1 c** upon excitation with femtosecond 640 nm light (see Figure S10 in the Supporting Information). As the closed isomer does not comprise different conformers or other simultaneously excited isomers, no other long‐lived species corresponding to any triplet state were observed. A similar behavior was found for compound **2 c** (see Figure S13 in the Supporting Information). Upon initial femtosecond laser pulse excitation with 640 nm, a FC absorption band was formed and relaxed fast (*τ*=170 fs) towards the singlet and also rather fast towards the ground state and some open isomer with a decay time of 1.5 ps. No contribution, neither from long‐lived bands nor from the electronically separated biacetyl moiety, was observed. The isolated biacetyl presumably keeps its original spectral features with a forbidden transition at the blue edge of the visible range. The quantitative conversion to the closed isomer **2 c** at 405 nm despite the reasonable quantum yield of ring‐opening at 577 nm (Table 1) might be a result of the different nature for orbitals excited upon irradiating into another absorption band of **2 c** at 405 nm.

The overall excitation pathways derived from the transient absorption measurements of DAEs **1** and **2** are summarized in Scheme 2. Except for the specific decay times, the main difference between these two derivatives arises from their major cyclization pathways, which occurs via the triplet manifold for **1 o** carrying the terminally attached biacetyl units but via the singlet manifold for **2 o** with an inner biacetyl unit. The reason for this might be the rotation of the inner biacetyl unit out of the plane of the thiophene moiety in **2 o** due to steric hindrance and a lower energetic coupling of the orbitals similarly to the reason mentioned for the blue‐shifted absorption as compared to **1 o**. Therefore, irradiation is leading to a localized excitation at the hexatriene scaffold, the ISC is suppressed at least in the antiparallel conformation, and ring‐closure occurs from the singlet manifold as usual for DAEs.

## Conclusions

The spectroscopic features of two DAE photoswitches bearing biacetyl triplet sensitizers were investigated in detail using stationary and transient spectroscopy in the nanosecond to microsecond as well as the femtosecond to picosecond range. We could show that establishing π‐conjugation between the photoswitchable hexatriene core and the biacetyl triplet sensitizer does not necessarily lead to a switching via the triplet excited state, which is known to prevent byproduct formation. On the contrary, the decoupling of the sensitizer by converting the open to the closed isomer can drastically increase the efficiency of the ring opening, which is originating from the singlet excited state in both investigated switches.

## Experimental Section

Synthetic procedures and characterization data including stationary and transient photochemistry are detailed in the Supporting Information.

## Conflict of interest

The authors declare no conflict of interest.

## Supporting information

As a service to our authors and readers, this journal provides supporting information supplied by the authors. Such materials are peer reviewed and may be re‐organized for online delivery, but are not copy‐edited or typeset. Technical support issues arising from supporting information (other than missing files) should be addressed to the authors.

Supplementary

## References

[1a] M. Irie , Chem. Rev. 2000, 100, 1685–1716; 11777416 10.1021/cr980069d

[1b] M. Irie , T. Fukaminato , K. Matsuda , S. Kobatake , Chem. Rev. 2014, 114, 12174–12277; 25514509 10.1021/cr500249p

[1c] M. Irie , M. Mohri , J. Org. Chem. 1988, 53, 803–808.

[2] B. Roubinet , M. Weber , H. Shojaei , M. Bates , M. L. Bossi , V. N. Belov , M. Irie , S. W. Hell , J. Am. Chem. Soc. 2017, 139, 6611–6620. 28437075 10.1021/jacs.7b00274

[3a] M. Irie , Y. Yokoyama , T. Seki , New Frontiers in Photochromism, Springer, Japan, 2013, p;

[3b] S. L. Gilat , S. H. Kawai , J. M. Lehn , Chem. Eur. J. 1995, 1, 275–284;

[3c] T. Shizuka , K. Seiya , K. Tsuyoshi , I. Masahiro , Chem. Lett. 2003, 32, 892–893.

[4a] E. Orgiu , P. Samorì , Adv. Mater. 2014, 26, 1827–1845; 24554562 10.1002/adma.201304695

[4b] M. E. Gemayel , K. Börjesson , M. Herder , D. T. Duong , J. A. Hutchison , C. Ruzié , G. Schweicher , A. Salleo , Y. Geerts , S. Hecht , E. Orgiu , P. Samorì , Nat. Commun. 2015, 6, 6330; 25739864 10.1038/ncomms7330

[4c] B. Kang-Jun , B. Maddalena , N. Dario , C. Mario , N. Yong-Young , Adv. Mater. 2013, 25, 4267–4295;23483718

[4d] R. Hayakawa , K. Higashiguchi , K. Matsuda , T. Chikyow , Y. Wakayama , ACS Appl. Mater. Interfaces 2013, 5, 3625–3630. 23548076 10.1021/am400030z

[5a] S. Kawata , Y. Kawata , Chem. Rev. 2000, 100, 1777–1788; 11777420 10.1021/cr980073p

[5b] R. C. Shallcross , P. Zacharias , A. Köhnen , P. O. Körner , E. Maibach , K. Meerholz , Adv. Mater. 2013, 25, 469–476. 23427339 10.1002/adma.201202186

[6a] T. Fukaminato , T. Hirose , T. Doi , M. Hazama , K. Matsuda , M. Irie , J. Am. Chem. Soc. 2014, 136, 17145–17154; 25390547 10.1021/ja5090749

[6b] S. Fredrich , R. Göstl , M. Herder , L. Grubert , S. Hecht , Angew. Chem. Int. Ed. 2016, 55, 1208–1212; 10.1002/anie.201509875 26662470

[7a] R. Göstl , B. Kobin , L. Grubert , M. Pätzel , S. Hecht , Chem. Eur. J. 2012, 18, 14282–14285; 23033200 10.1002/chem.201203111

[7b] R. Li , T. Nakashima , O. Galangau , S. Iijima , R. Kanazawa , T. Kawai , Chem. Asian J. 2015, 10, 1725–1730. 26097224 10.1002/asia.201500328

[8] M. Herder , F. Eisenreich , A. Bonasera , A. Grafl , L. Grubert , M. Pätzel , J. Schwarz , S. Hecht , Chem. Eur. J. 2017, 23, 3743–3754. 28093831 10.1002/chem.201605511

[9a] M. Irie , T. Lifka , K. Uchida , S. Kobatake , Y. Shindo , Chem. Commun. 1999, 747–750;

[9b] H. Kenji , M. Kenji , K. Seiya , Y. Taro , K. Tsuyoshi , I. Masahiro , Bull. Chem. Soc. Jpn. 2000, 73, 2389–2394.

[10a] Y.-C. Jeong , S. I. Yang , E. Kim , K.-H. Ahn , Tetrahedron 2006, 62, 5855–5861;

[10b] Y.-C. Jeong , D. G. Park , I. S. Lee , S. I. Yang , K.-H. Ahn , J. Mater. Chem. 2009, 19, 97–103.

[11a] M. Boggio-Pasqua , M. Ravaglia , M. J. Bearpark , M. Garavelli , M. A. Robb , J. Phys. Chem. A 2003, 107, 11139–11152;

[11b] A. Perrier , S. Aloise , M. Olivucci , D. Jacquemin , J. Phys. Chem. Lett. 2013, 4, 2190–2196;

[11c] S. Nakamura , K. Uchida , M. Hatakeyama , Molecules 2013, 18, 5091; 23644976 10.3390/molecules18055091 PMC6270092

[11d] P. D. Patel , I. A. Mikhailov , K. D. Belfield , A. E. Masunov , Int. J. Quantum Chem. 2009, 109, 3711–3722.

[12a] I. Hamdi , G. Buntinx , A. Perrier , O. Devos , N. Jaidane , S. Delbaere , A. K. Tiwari , J. Dubois , M. Takeshita , Y. Wada , S. Aloise , Phys. Chem. Chem. Phys. 2016, 18, 28091–28100; 27711399 10.1039/c6cp03471c

[12b] S. Aloïse , R. Yibin , I. Hamdi , G. Buntinx , A. Perrier , F. Maurel , D. Jacquemin , M. Takeshita , Phys. Chem. Chem. Phys. 2014, 16, 26762–26768; 25372933 10.1039/c4cp03641g

[12c] Y. Ishibashi , M. Fujiwara , T. Umesato , H. Saito , S. Kobatake , M. Irie , H. Miyasaka , J. Phys. Chem. C 2011, 115, 4265–4272;

[12d] E. Pontecorvo , C. Ferrante , C. G. Elles , T. Scopigno , J. Phys. Chem. B 2014, 118, 6915–6921; 24886226 10.1021/jp5051047

[12e] H. Jean-Ruel , R. R. Cooney , M. Gao , C. Lu , M. A. Kochman , C. A. Morrison , R. J. D. Miller , J. Phys. Chem. A 2011, 115, 13158–13168. 21939249 10.1021/jp205818h

[13a] R. Murata , T. Yago , M. Wakasa , Bull. Chem. Soc. Jpn. 2011, 84, 1336–1338;

[13b] R. Murata , T. Yago , M. Wakasa , J. Phys. Chem. A 2015, 119, 11138–11145; 26490486 10.1021/acs.jpca.5b08205

[13c] K. Tani , Y. Ishibashi , H. Miyasaka , S. Kobatake , M. Irie , J. Phys. Chem. C 2008, 112, 11150–11157;

[13d] M. T. Indelli , S. Carli , M. Ghirotti , C. Chiorboli , M. Ravaglia , M. Garavelli , F. Scandola , J. Am. Chem. Soc. 2008, 130, 7286–7299; 18479107 10.1021/ja711173z

[13e] R. T. F. Jukes , V. Adamo , F. Hartl , P. Belser , L. De Cola , Inorg. Chem. 2004, 43, 2779–2792. 15106964 10.1021/ic035334e

[14] A. Thomas Bens , D. Frewert , K. Kodatis , C. Kryschi , H. D. Martin , H. P. Trommsdorff , Eur. J. Org. Chem. 1998, 2333–2338.

[15] C. S. Foote , Science 1968, 162, 963–970. 4972417 10.1126/science.162.3857.963

[16a] K. Uchida , Y. Nakayama , M. Irie , Bull. Chem. Soc. Jpn. 1990, 63, 1311–1315;

[16b] M. Irie , O. Miyatake , K. Uchida , J. Am. Chem. Soc. 1992, 114, 8715–8716;

[16c] Y. Ishibashi , T. Umesato , M. Fujiwara , K. Une , Y. Yoneda , H. Sotome , T. Katayama , S. Kobatake , T. Asahi , M. Irie , H. Miyasaka , J. Phys. Chem. C 2016, 120, 1170–1177.

[17a] K. Uchida , E. Tsuchida , Y. Aoi , S. Nakamura , M. Irie , Chem. Lett. 1999, 28, 63–64;

[17b] K. Uchida , D. Guillaumont , E. Tsuchida , G. Mochizuki , M. Irie , A. Murakami , S. Nakamura , J. Mol. Struct. 2002, 579, 115–120.

[18a] C. Franco , J. Olmsted , Talanta 1990, 37, 905–909; 18965040 10.1016/0039-9140(90)80251-a

[18b] M. Quaranta , M. Murkovic , I. Klimant , Analyst 2013, 138, 6243–6245. 23803965 10.1039/c3an36782g

[19] J. Woodhouse , G. Nass Kovacs , N. Coquelle , L. M. Uriarte , V. Adam , T. R. M. Barends , M. Byrdin , E. de la Mora , R. Bruce Doak , M. Feliks , M. Field , F. Fieschi , V. Guillon , S. Jakobs , Y. Joti , P. Macheboeuf , K. Motomura , K. Nass , S. Owada , C. M. Roome , C. Ruckebusch , G. Schirò , R. L. Shoeman , M. Thepaut , T. Togashi , K. Tono , M. Yabashi , M. Cammarata , L. Foucar , D. Bourgeois , M. Sliwa , J.-P. Colletier , I. Schlichting , M. Weik , Nat. Commun. 2020, 11, 741. 32029745 10.1038/s41467-020-14537-0 PMC7005145

[20] S. Aloïse , C. Ruckebusch , L. Blanchet , J. Réhault , G. Buntinx , J.-P. Huvenne , J. Phys. Chem. A 2008, 112, 224–231. 18154275 10.1021/jp075829f

