# Supplementary material for: Mechanistic Insights into the Triplet Sensitized Photochromism of Diarylethenes
Source: Chemistry. 2020 May 26;26(34):7672–7. doi: 10.1002/chem.202000877 (PMC7318355; doi:10.1002/chem.202000877)
Supplement: Supplementary file 1 — Supplementary [file CHEM-26-7672-s001.pdf]

Chemistry–A European Journal

Supporting Information

Mechanistic Insights into the Triplet Sensitized Photochromism of Diarylethenes

Sebastian Fredrich,[a] Tobias Morack,[a] Michel Sliwa,[b] and Stefan Hecht*[a, c, d]

Contents

1. General Methods .... S 2

2. Synthesis .... S 3

3. Photochemical Features .... S 8

3.1. Determination of quantum yields and actinometry .... S 8

3.2. Switching cycles and fatigue resistance .... S 10

3.3. Transient spectroscopy data .... S 11

3.4. Decay traces and Quenching experiments with carotene .... S 19

3.5. Excitation Schemes .... S 20

3.6. Fluorescence spectra of **2c** .... S 22

4. NMR-Spectra of **2** .... S 23

1. General Methods

Chemicals were purchased at Sigma Aldrich or ABCR and used without further purification. Solvents were distilled and dried or degassed if necessary before use. For spectroscopy experiments solvents of spectroscopic grade were used. For column chromatography silica gel (0.035-0.070 mm, 60 Å) was used.

NMR-spectra were recorded at a Bruker AVANCE II 500 spectrometer (^{1}H: 500 MHz, ^{13}C: 125 MHz) or at a Bruker AVANCE 300 (^{1}H: 300 MHz, ^{13}C: 75 MHz). The samples were dissolved in deuterated solvents, using residual proton signals as standard (^{1}H: δ($CHCl_3$ = 7.26 ppm, $CHDCl_2$ = 5.32 ppm), (^{13}C: δ($CDCl_3$ = 77.16 ppm, CD_2Cl_2 = 53.84 ppm). Chemical shifts are denoted in δ (ppm), coupling constants J in Hz. The splitting patterns are designated as: s (singlet); d (doublet); t (triplet); q (quartet); quin (quintet); m (multiplet). Ultra-high performance liquid chromatography electro-spray ionization mass spectrometry (UPLC-ESI-MS) was performed on a Waters Acquity UPLC (gradient mixtures of acetonitrile/water) with a Waters LCT Premier XE mass detector. Additionally a Waters Alliance System with Waters Separations Module 2695, a Waters Diode Array Detector 996 and a Waters Mass Detector ZQ 2000 were used. For the diarylethene samples **1**, **2** and **3** the chromatographic separation was performed with a gradient of 5% to 95% acetonitrile in water with 0.1% formic acid. To determine the purity of these compounds the integration of the UV/vis-trace from 210 nm to 800 nm was used.

Stationary UV-vis-spectra were recorded using quartz cuvettes on a Varian Cary 50 or a Varian Cary 60 spectrophotometer with Peltier thermostated cell holder at 25 ± 0.1 °C.

For quantitative irradiation and quantum yield determination experiments an Oriel 500 W Hg(Xe)-short-arc lamp model LSB740 in an Oriel arc lamp box LSH302 was used. It was equipped with an Oriel 300 mm grating monochromator model MSH-300, a shutter and a light guide by Oriel. Light intensities were usually between 5×10^{9} and 1×10^{11} photons $s^{-1}cm^{-1}$.

Long term irradiation was realized with a 1000 W high pressure Xe lamp, an interference filter (Asahi Spectra, 50 % transmission at λ_{max} = 310 nm, FWHM = 10 nm) and several cut-off filters. Light intensities were not determined but are generally higher than for the Oriel 500 W.

2. Synthesis

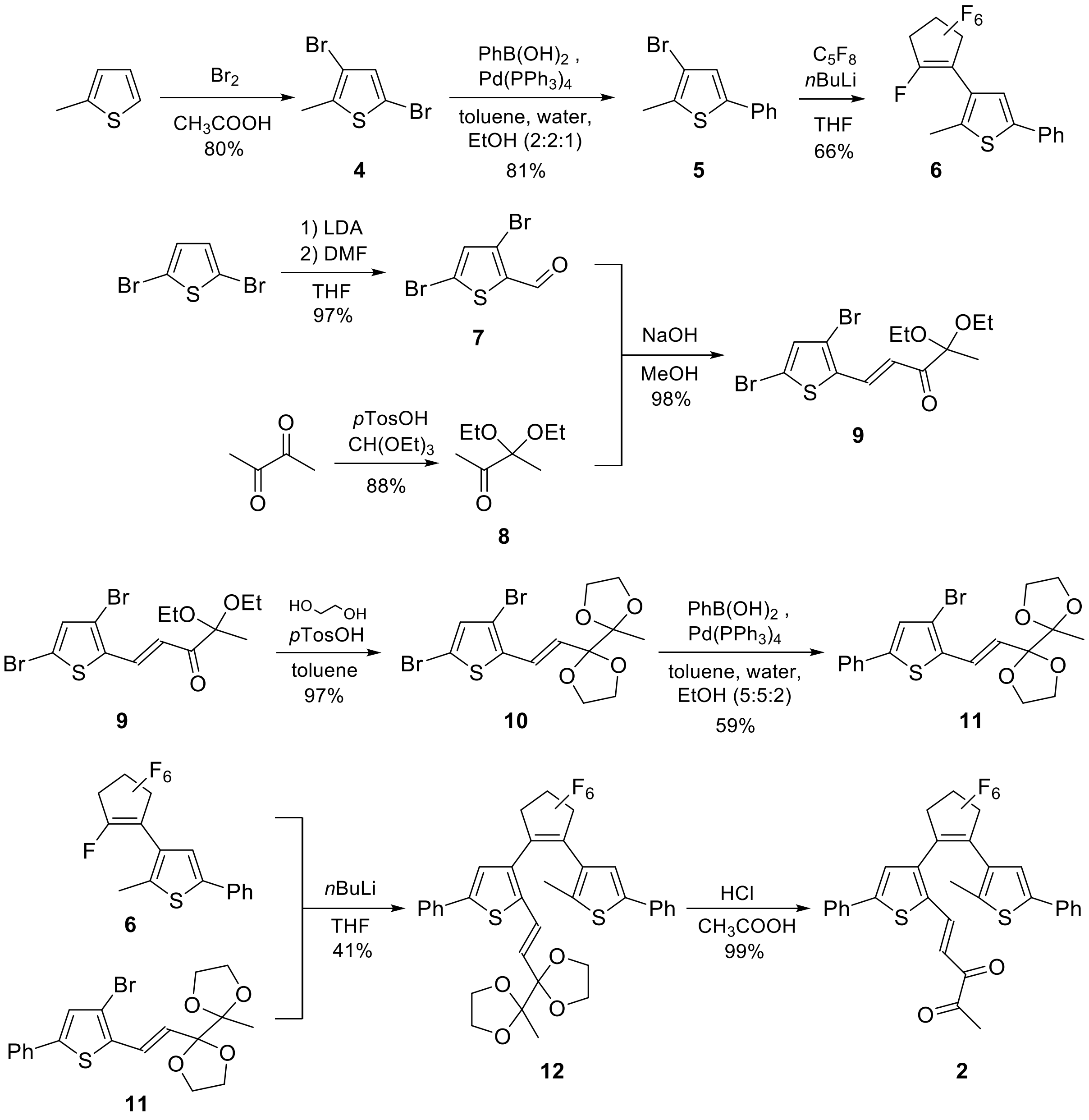

Scheme S1: General scheme for the synthesis of compound **2**

The synthesis of the investigated compounds **1** and **3** as well as the precursor molecules **4-8** was already described.[1]

---

[1] a) S. Fredrich, R. Göstl, M. Herder, L. Grubert and S. Hecht, *Angew. Chem. Int. Ed.* **2016**, *55*, 1208-1212; b) M. Irie, T. Lifka, S. Kobatake and N. Kato, *J. Am. Chem. Soc.* **2000**, *122*, 4871-4876; c) D. Sud, J. Wigglesworth Tony and R. Branda Neil *Angew. Chem. Int. Ed.* **2007**, *46*, 8017-8019; d) V. Valderrey, A. Bonasera, S. Fredrich and S. Hecht, *Angew. Chem. Int. Ed.* **2017**, *56*, 1914-1918.

(*E*)-1-(3,5-dibromothiophen-2-yl)-4,4-diethoxypent-1-en-3-one (9)

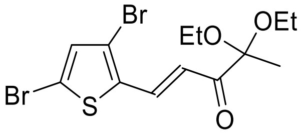

3,5-Dibromothiophene-2-carbaldehyde **7** (11.51 g, 42.64 mmol, 1.1 eq.) und 3,3-Diethoxybutan-2-one **8** (6.21 g, 38.76 mmol, 1.0 eq.) were dissolved in MeOH. To this mixture 60 ml 20% aqueous NaOH-solution was added and stirred at room temperature for five days. The MeOH was distilled off and the aqueous phase was extracted with ethyl acetate. The organic phase was washed with saturated NaCl-solution and dried over $MgSO_4$. After evaporation of the solvent, column chromatography (silica, petroleum ether : ethyl acetate 20:1) gave the product (15.64 g, 37.95 mmol, 98%) as light brown solid.

^{1}H-NMR (300 MHz, $CDCl_3$): δ (ppm) = 7.80 (d, 1H, ^3J(H,H) = 15.8 Hz), 7.03 (s, 1H), 7.01 (d, 1H, ^3J(H,H) = 15.8 Hz), 3.52 (m, 4H), 1.44 (s, 3H), 1.24 (t, 6H, ^3J(H,H) = 7.1 Hz).

^{13}C-NMR (75 MHz, $CDCl_3$): δ (ppm) = 196.8, 136.7, 134.0, 133.8, 121.5, 116.6, 116.0, 102.1, 57.8, 21.0, 15.4.

(*E*)-2-(2-(3,5-dibromothiophen-2-yl)vinyl)-2'-methyl-2,2'-bi(1,3-dioxolane) (10)

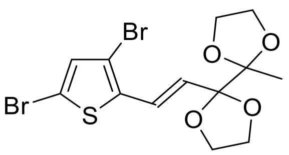

Compound **9** (15.64 g, 37.95 mmol, 1.0 eq.) was dissolved in 200 ml toluene and 100 ml ethylene glycol (1.788 mol, 47.3 eq.), as well as *p*-toluene sulfonic acid monohydrate (2.17 g, 11.39 mmol, 0.3 eq.) were added. The reaction mixture was heated to reflux using a Dean-Stark-trap for 2 h. It was washed with water and saturated NaCl-solution and extracted with ethyl acetate. The organic phase was dried over $MgSO_4$ and evaporated under reduced pressure. The product was obtained as a light brown liquid (15.76 g, 36.99 mmol, 97%) that was used in the next step without further purification.

(*E*)-2-(2-(3-bromo-5-phenylthiophen-2-yl)vinyl)-2'-methyl-2,2'-bi(1,3-dioxolane) (11)

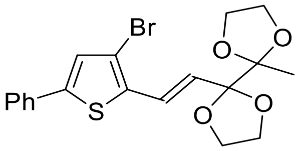

10 (1.24 g, 2.91 mmol, 1.0 eq.) was dissolved in 15 ml toluene and 15 ml 2 M aqueous Na_2CO_3-solution and degassed by flushing with argon. Phenyl boronic acid (0.36 g, 2.91 mmol, 1.0 eq.) was separately dissolved in 6 ml EtOH and degassed likewise. Both solutions were combined and $Pd(PPh_3)_4$ (0.17 g, 0.15 mmol, 0.05 eq.) was added. The reaction mixture was heated to reflux for 17 h. It was subsequently extracted with ethyl acetate. The organic phase was washed with water, dried over $MgSO_4$ and evaporated under reduced pressure. The product was purified via column chromatography (silica, petroleum ether : ethyl acetate 4:1) and recrystallized from EtOH to give a yellow solid (0.73 g, 1.72 mmol, 59%).

^{1}H-NMR (300 MHz, $CDCl_3$): δ (ppm) = 7.55 (m, 2H), 7.42 - 7.32 (m, 3H), 7.17 (s, 1H), 7.14 (d, 1H, ^{3}J(H,H) = 15.9 Hz), 6.51 (d, 1H, ^{3}J(H,H) = 15.9 Hz), 4.18 - 4.09 (m, 4H), 3.92 – 3.75 (m, 4H), 1.46 (s, 3H).

^{13}C-NMR (75 MHz, $CDCl_3$): δ (ppm) = 143.0, 135.0, 132.9, 129.0, 128.4, 126.4, 125.5, 125.1, 124.6, 112.5, 95.2, 93.6, 61.3, 61.1, 20.5.

(*E*)-2-(2-(3-(3,3,4,4,5,5-hexafluoro-2-(2-methyl-5-phenylthiophen-3-yl)cyclopent-1-en-1-yl)-5-phenylthiophen-2-yl)vinyl)-2'-methyl-2,2'-bi(1,3-dioxolane) (12)

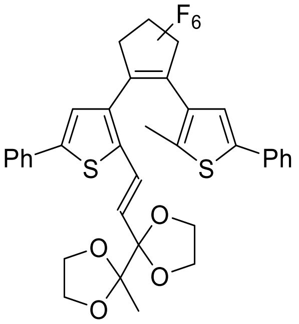

11 (1.03 g, 2.43 mmol, 1.0 eq.) was dissolved in 20 ml dry degassed THF and cooled to -90°C. To this solution *n*-butyllithium (2.2 M in *c*-hexane, 1.33 ml, 2.92 mmol, 1.2 eq.) was slowly added and the mixture was stirred for 5 min. **6** (0.89 g, 2.43 mmol, 1.0 eq.) was separately dissolved in 10 ml

dry degassed THF and cooled to -90°C as well. The first solution was added to the second and the resulting mixture was further stirred for 30 min at low temperature and then allowed to warm to room temperature. Water was added and it was extracted with ethyl acetate. The organic phase was washed with 1 M HCl, dried over $MgSO_4$ and evaporated under reduced pressure. It was purified via column chromatography (silica, petroleum ether : ethyl acetate 4:1) and the product (0.68 g, 0.99 mmol, 41%) was obtained as white solid.

^{1}H-NMR (300 MHz, $CDCl_3$): δ (ppm) = 7.61 - 7.50 (m, 6H), 7.44 – 7.27 (m, 12H), 6.60 (d, 1H, ^{3}J(H,H) = 15.6 Hz), 6.49 (d, 0.5H, ^{3}J(H,H) = 15.6 Hz), 6.41 (d, 1H, ^{3}J(H,H) = 15.6 Hz), 6.14 (d, 0.5H, ^{3}J(H,H) = 15.6 Hz), 3.99 - 3.79 (m, 7H), 3.62 - 3.56 (m, 3H), 3.37 - 3.32 (m, 2H), 2.00 (s, 1.5H), 1.98 (s, 3H), 1.14 (s, 1.5H), 1.08 (s, 3H).

^{13}C-NMR (75 MHz, $CDCl_3$): δ (ppm) = 144.0, 143.8, 141.9, 141.7, 141.2, 141.1, 133.5, 133.3, 133.0, 132.9, 129.3, 129.1, 128.9, 128.5, 128.4, 127.8, 127.8, 127.3, 126.0, 125.8, 125.4, 125.3, 123.2, 123,1 123.0, 122.7, 121.8, 110.3, 109.4, 94.9, 92.9, 66.0, 65.7, 61.1, 60.7, 20.5, 20.0, 14.8, 14.5, 14.2.

(*E*)-5-(3-(3,3,4,4,5,5-hexafluoro-2-(2-methyl-5-phenylthiophen-3-yl)cyclopent-1-en-1-yl)-5-phenylthiophen-2-yl)pent-4-ene-2,3-dione (2)

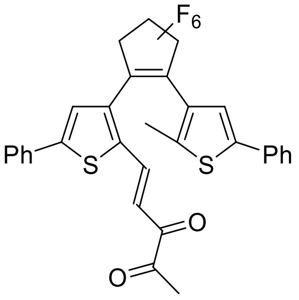

Compound **12** (680 mg, 0.984 mmol, 1.0 eq.) was dissolved in 20 ml glacial acetic acid and 4.5 ml HCl were added. The mixture was heated to reflux for 5 h. After cooling to room temperature, water was added and the mixture was extracted with ethyl acetate. The organic phase was washed with saturated NaCl-solution, dried over $MgSO_4$ and evaporated under reduced pressure. The product was recrystallized from water/EtOH (1:1) and the pure compound was obtained as light yellow solid (590 mg, 0.979 mmol, 99%).

^{1}H-NMR (300 MHz, $CDCl_3$): δ (ppm) = 7.64 (m, 2H), 7.44 (m, 6H), 7.36 (m, 3H), 7.29 (m, 1H), 7.07 (s, 1H), 5.68 (s, 1H), 2.02 (s, 3H), 1.31 (d, 3H, ^{3}J(H,H) = 7.2 Hz).

^{13}C-NMR (75 MHz, $CDCl_3$): δ (ppm) = 203.7, 176.8, 150.4, 142.4, 133.0, 131.9, 130.7, 130.3, 129.7, 129.4, 129.1, 128.0, 126.2, 125.6, 124.9, 124.8, 122.2, 101.8, 82.4, 13.2, 14.4.

^{19}F-NMR (104 MHz, $CDCl_3$): δ (ppm) = -110.4 (s, 2F), -111.7 (d, 2F, J = 68 Hz), -132.4 (d, 2F, J = 28 Hz).

MS(ESI^{+}): m/z 603.08 (calcd: 603.09 for $[C_{31}H_{21}F_6S_2O_2]^{+}$)

3. Photochemistry

3.1. Determination of quantum yields and actinometry

Quantum yields for ring-closure reactions with UV-light were determined using the initial slope method. Light intensity at 289, 313, 365 and 405 nm was determined by using ferrioxalate actinometry.[2] For this purpose, 3 ml of a 0.006 M solution in 0.05 M H_2SO_4 was irradiated and 0.5 ml of phenanthroline (0.1 wt% in 0.5 M H_2SO_4/1.6 M NaOAc) were added subsequently. The resulting absorbance at 510 nm was used to calculate the light intensity via Formula S1.

$$I_0 = \frac{\Delta A_{510nm}}{\Delta t * \varepsilon_{510nm} * \Phi_{\mathrm{irr}} * 1000} * \frac{3.5mL}{3mL} \qquad \textbf{(S1)}$$

Thereby $\Delta A_{510\mathrm{nm}}$ is the difference of the absorption at 510 nm for an irradiated versus a non-irradiated solution, Δt is the irradiation time, $\varepsilon_{510\mathrm{nm}}$ is 11100 M^{-1} cm^{-1} and Φ_{irr} is the quantum yield at the used irradiation wavelength (1.24 for 289 nm, 1.24 for 313 nm, 1.26 for 365 nm, 1.14 for 405 nm).[3]

For the determination of the quantum yield Formula S2 was used:

$$\Phi = \frac{\Delta A / \Delta t}{(1 - 10^{-A'}) * \varepsilon * I_0 * 1000} \qquad \textbf{(S2)}$$

for which $\Delta A/\Delta t$ is the change of absorbance at a wavelength, which changes upon irradiation within the time. $1 - 10^{-A'}$ is the percentage of the absorbed photons by the solution at irradiation wavelength, ε is the extinction coefficient at observed wavelength and I_0 the light intensity. This method of calculating the quantum yield was applied for the initial stages of the reaction (maximum 5% of photochemical conversion).

[2] C. G. Hatchard and C. A. Parker, *Proc. Roy. Soc. (London) A* **1956**, *235*, 518-536.

[3] M. Montalti, A. Credi, L. Prodi and M. T. Gandolfi, *Handbook of Photochemistry, Third Edition*, CRC Press: Boca Raton, **2006.**

For the ring-opening a wavelength was needed that exceed the range for ferrioxalate actinometry. Therefore actinometry via aberchrome 670 was performed.[4] A toluene solution (1.0·10-4 M) was first irradiated with 365 nm. The formed isomer was irradiated back with 577 nm. The decay of the band at 519 nm indicates the light intensity via formula S 3:

$$I_0 = -\frac{\Delta A_{519nm}}{\left(1 - 10^{-A'}\right) * \varepsilon_{519nm} * \Delta t * \Phi_{irr} * 1000} \qquad \textbf{(S3)}$$

for which $\Delta A_{519\mathrm{nm}}$ is the difference of the absorption at 519 nm for an irradiation interval Δt. $\varepsilon_{519\mathrm{nm}}$ is 7760 $M^{-1}cm^{-1}$ and Φ_{irr} is the quantum yield at the used irradiation wavelength 577 nm (0.28).

The ratio of open and closed isomer at the photostationary state (PSS) was determined by injection of irradiated samples into a UPLC and detection with and UV/vis diode array integrated at the isosbestic point of the corresponding irradiation spectrum. The different ratio of water and acetonitrile caused by the gradient in the UPLC was disregarded as no significant shift of the spectra compared to the pure acetonitrile solutions was observed.

For oxygen dependent measurements, the samples were carefully degassed using freeze-pump-thaw technique two times. Therefore, a cuvette with a stopcock was applied. The sample was frozen in liquid nitrogen, vacuum was applied during the melting process and the cuvette was refilled with argon subsequently. The procedure was repeated twice to exclude oxygen almost completely from the measurement.

[4] A. P. Glaze, H. G. Heller and J. Whittall, *J. Chem. Soc. Perk. Trans. 2* **1992**, 591-594.

3.2. Switching cycles and fatigue resistance

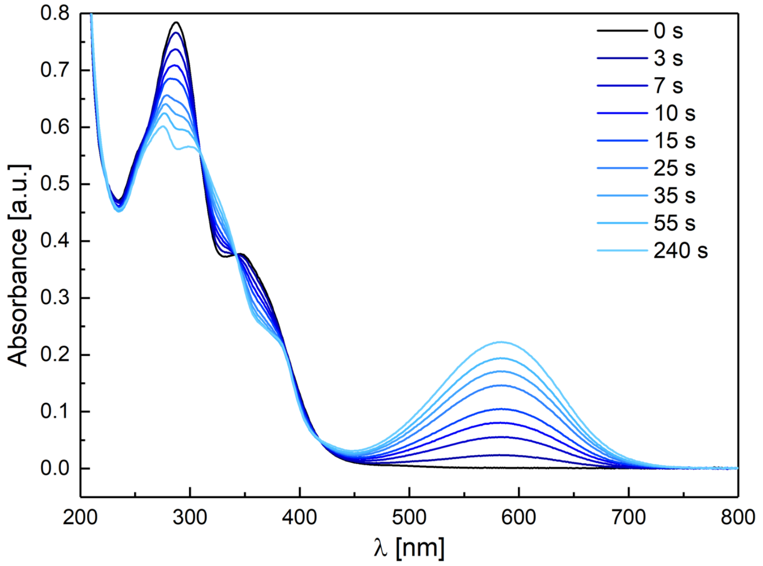

Figure S1: Absorption spectra of **2** in CH_3CN ($4{\cdot}10^{-5}$ M, 25°C) upon irradiation with 365 nm.

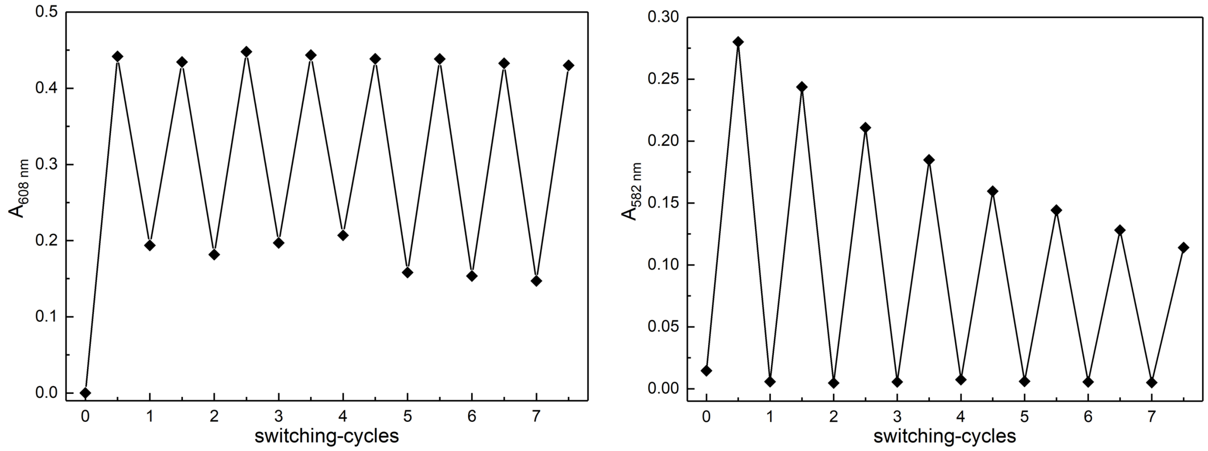

Figure S2: Switching cycles consisting of ring-closure and ring-opening obtained by alternate irradiation with 405 nm and >500 nm in degassed CH_3CN solutions irradiated with a 1000 W Xe lamp for: left: **1** indicated by the maximum of the closed form at 608 nm; right: **2** indicated by the maximum of the closed form at 582 nm.

3.3. Transient spectroscopy data

The nanosecond transient absorption spectroscopy setup has been described elsewhere.[5] A third harmonic pulse (355 nm) of a Nd:YAG laser with an output power of ca. 1 mJ and a pulse width of 5 ns was used as excitation beam and a pulsed Xe lamp was utilized as probe light. Transient difference absorption spectra were obtained from the transient absorption decays recorded in steps of 10 nm wavelengths as absorbance changes for the given delay-times.

Femtosecond time-resolved experiments were performed with a pump–probe spectrometer[6] based on a Ti:sapphire laser system (Coherent Mira-900-D oscillator and Libra-S regenerative amplifier) delivering 800 nm, 1 mJ and 90 fs pulses with a repetition rate of 1 kHz. The pump pulses at 320 nm, 400 nm and 640 nm were generated by frequency multiplying the output of a Quantronix Palitra OPA pumped at 800 nm with an energy at the sample of about 2 μJ (0.2 mJ cm^{-2}). The probe pulses were obtained by focusing 1 mJ, 800 nm pulses into a 1 mm CaF_2 plate to generate a white light continuum. The pump–probe polarization configuration was set at the magic angle (54.7°). Transient absorbance was obtained by comparing signal and reference spectra with and without pump pulses for different delay-times. The delay-times between the pump and the probe were varied up to 1 ns using an optical delay line. Sample solutions (OD ~ 1 at the pump wavelength) were circulated in a flow-cell equipped with a 200 mm thick CaF_2 entrance window and an optical path length of 2 mm. The reservoir was continuously irradiated by visible or UV light to avoid photoproduct accumulation. All the transient spectra presented in this paper are GVD corrected according to the typical extrapolation method[7] (the temporal chirp over the white-light 350–800 nm range was about 400 fs). The characteristic times deduced from kinetics were obtained by fitting the data with the result of a multiexponential function convolved

[5] a) T. Stoll, M. Gennari, I. Serrano, J. Fortage, J. Chauvin, F. Odobel, M. Rebarz, O. Poizat, M. Sliwa, A. Deronzier, M.-N. Collomb, *Chem. Eur. J.* **2013**, *19*, 782-792; b) J. Woodhouse *et al.*, *Nat. Commun.* **2020**, *11*, 741.

[6] A. Tokunaga, L. Martinez Uriarte, K. Mutoh, E. Fron, J. Hofkens, M. Sliwa, J. Abe, *J. Am. Chem. Soc.* **2019**, *141*, *44*, 17744-17753

[7] a) M. Ziólek, M. Lorenc, R. Naskrecki, *Appl. Phys. B* **2001**, *72*, 843-847; b) T. Nakayama, Y. Amijima, K. Ibuki, K. Hamanoue, *Rev. Sci. Instrum.* **1997**, *68*, 4364-4371; C. Ruckebusch, M. Sliwa, P. Pernot, A. de Juan and R. Tauler, *J. Photochem. Photobiol. C* **2012**, *13*, 1-27.

using an initial Gaussian pulse for the pump–probe cross-correlation function (FWHM of 150 fs). For the sequence, variations were done according to a HS-MCR-method.

Spectrokinetic data were fitted with initial Gaussian pulses and subsequent decays. The spectra of the pure excited states (or mixtures of the parallel and antiparallel form, which could not be separated for most of the experiments) were extracted. The resulting model was checked and optimized according to the lack of fit (formula S4):

$$lack\ of\ fit = \sqrt{\frac{\sum_{i,j}(d_{i,j} - s_{i,j})^2}{\sum_{i,j} d_{i,j}^2}} \qquad \textbf{(S4)}$$

for which d are the measured data from the time i versus wavelength j matrix and s are the corresponding values for the simulated data according to the extracted spectra and excited states lifetimes. The lack of fit is given as percentage of deviation.

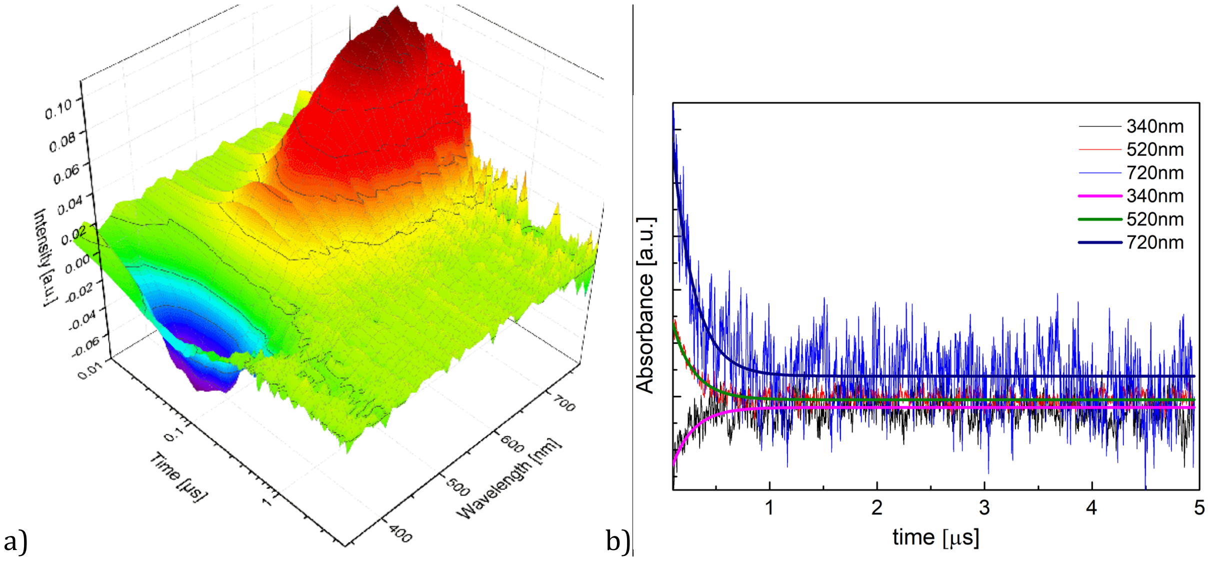

Figure S3: a) Spectra obtained of a solution of **1o** (non-degassed, CH_3CN, $4{\cdot}10^{-5}$ M, 25°C) upon irradiation with a 355 nm initial pulse in the nanosecond range until 5 µs; b) time profiles showing the decays of the absorbance at three different wavelengths. The fitted lines represent the modelled data as indicated in Table S1; the lack of fit was determined to be: 18.8 %.

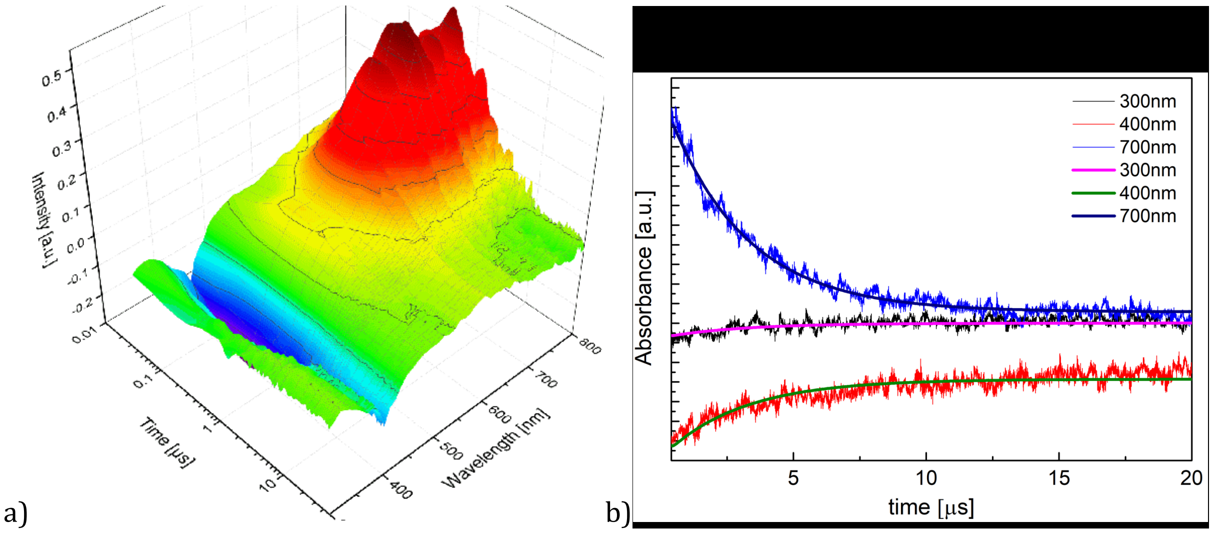

Figure S4: a) Spectra obtained of a solution of **1o** (degassed, CH_3CN, $4 \cdot 10^{-5}$ M, 25°C) upon irradiation with a 355 nm initial pulse in the nanosecond range until 50 µs; b) time profiles showing the decays of the absorbance at three different wavelengths. The fitted lines represent the modelled data as indicated in Table S1; the lack of fit was determined to be: 9.4 %.

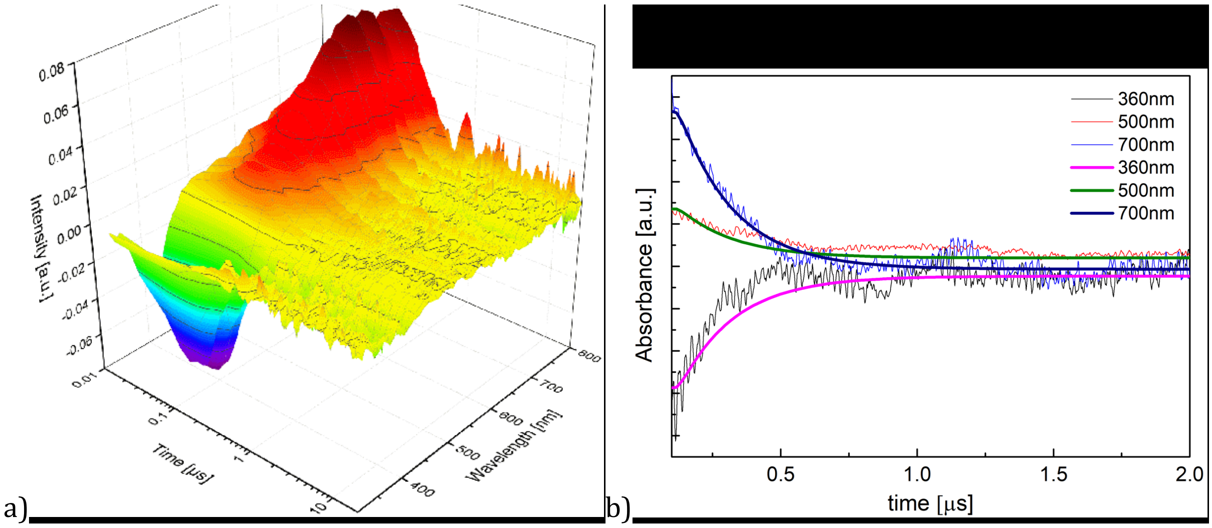

Figure S5: a) Spectra obtained of a solution of **1o** (flushed with oxygen, CH_3CN, $4 \cdot 10^{-5}$ M, 25°C) upon irradiation with a 355 nm initial pulse in the nanosecond range until 20 µs; b) time profiles showing the decays of the absorbance at three different wavelengths. The fitted lines represent the modelled data as indicated in Table S1; the lack of fit was determined to be: 32.9 %. The large deviation originates most likely from the noisy measurement.

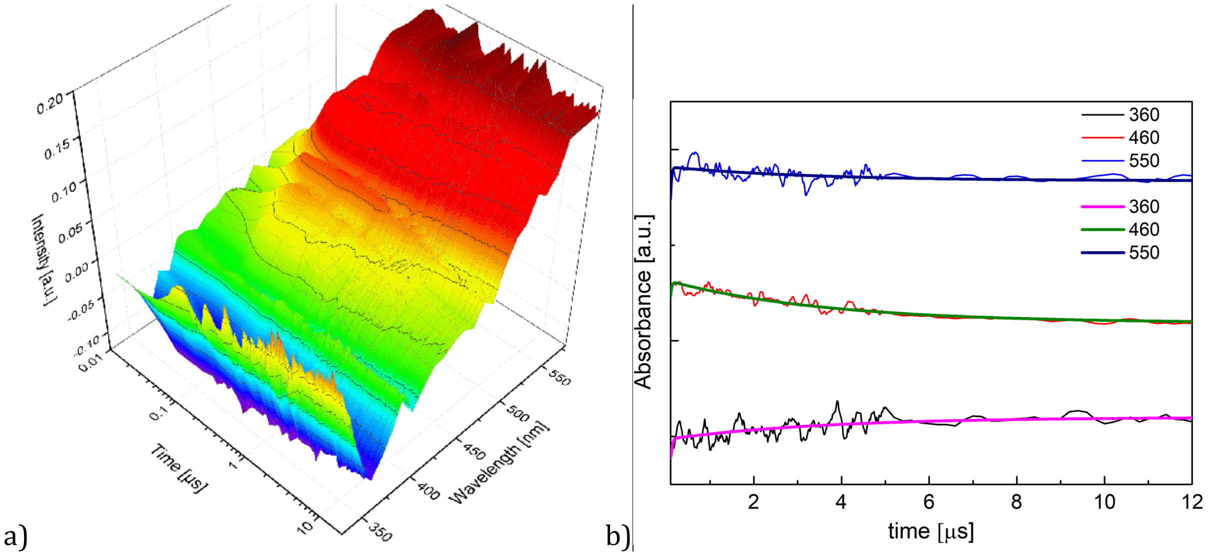

Figure S6: a) Spectra obtained of a solution of **2o** (degassed, CH_3CN, $4{\cdot}10^{-5}$ M, 25°C) upon irradiation with a 355 nm initial pulse in the nanosecond range until 20 µs; b) time profiles showing the decays of the absorbance at three different wavelengths. The fitted lines represent the modelled data as indicated in Table S1; the lack of fit was determined to be: 7.9 %.

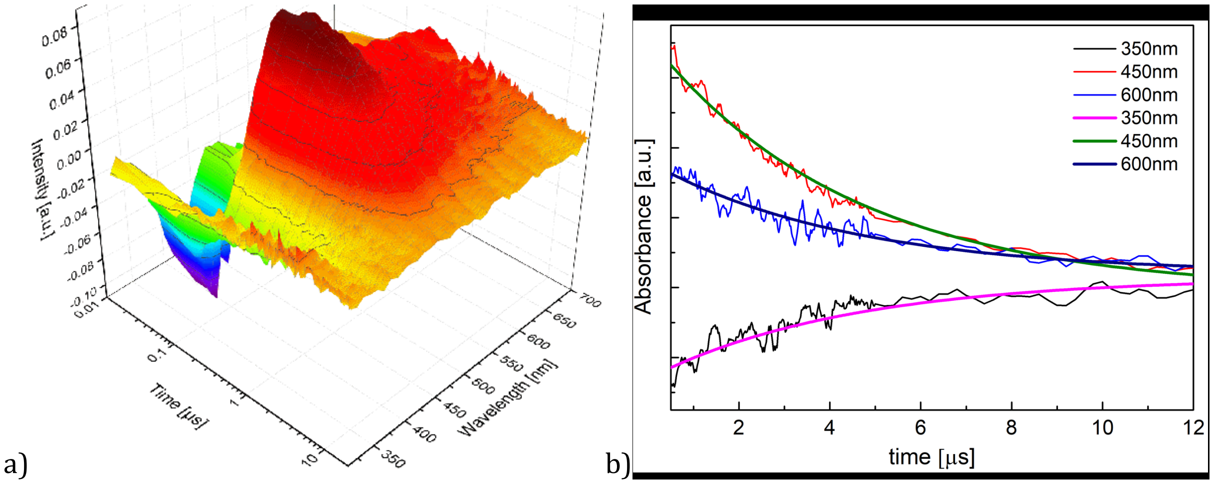

Figure S7: a) Spectra obtained of a solution of **2c** (degassed, CH_3CN, $4{\cdot}10^{-5}$ M, 25°C) upon irradiation with a 355 nm initial pulse in the nanosecond range until 20 µs; b) time profiles showing the decays of the absorbance at three different wavelengths. The fitted lines represent the modelled data as indicated in Table S1; the lack of fit was determined to be: 22.7 %. The large deviation originates most likely from fluorescence present already at the initial pulse (compare Figure S20).

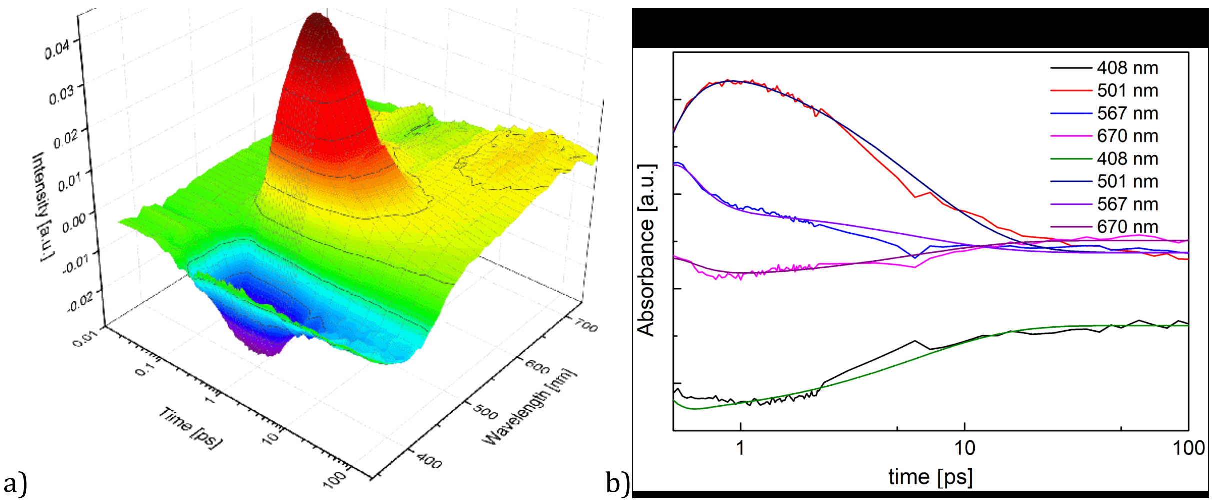

Figure S8: a) Spectra obtained of a solution of **1o** (non-degassed, CH_3CN, $4{\cdot}10^{-5}$ M, 25°C) upon irradiation with a 320 nm initial pulse in the femtosecond range until 200 ps; b) time profiles showing the decays of the absorbance at three different wavelengths. The fitted lines represent the modelled data as indicated in Table S1; the lack of fit was determined to be: 5.5 %.

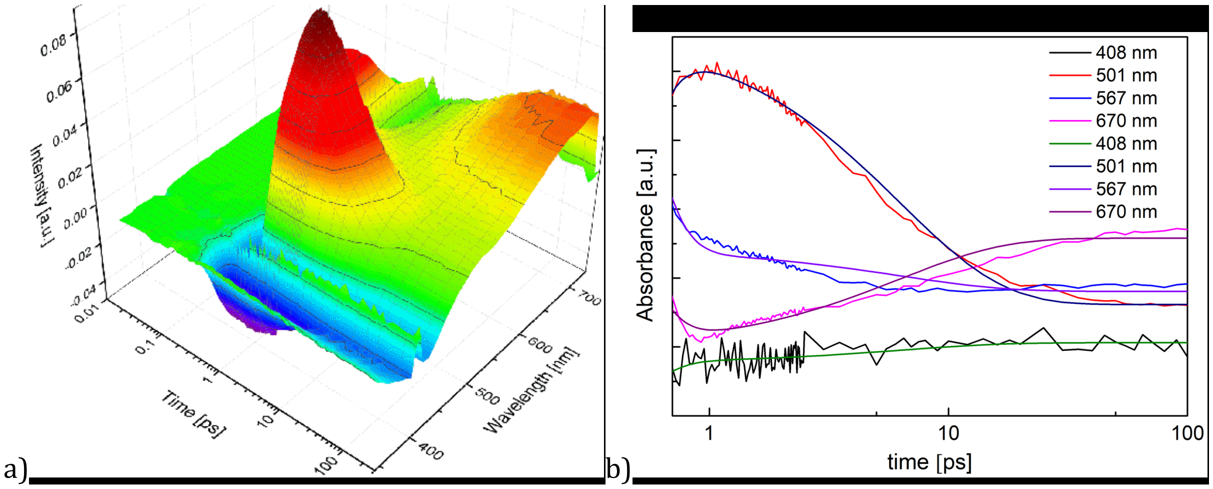

Figure S9: a) spectra obtained of a solution of **1o** (non-degassed, CH_3CN, $4{\cdot}10^{-5}$ M, 25°C) upon irradiation with a 400 nm initial pulse in the femtosecond range until 300 ps; b) time profiles showing the decays of the absorbance at three different wavelengths. The fitted lines represent the modelled data as indicated in Table S1; the lack of fit was determined to be: 10.0 %.

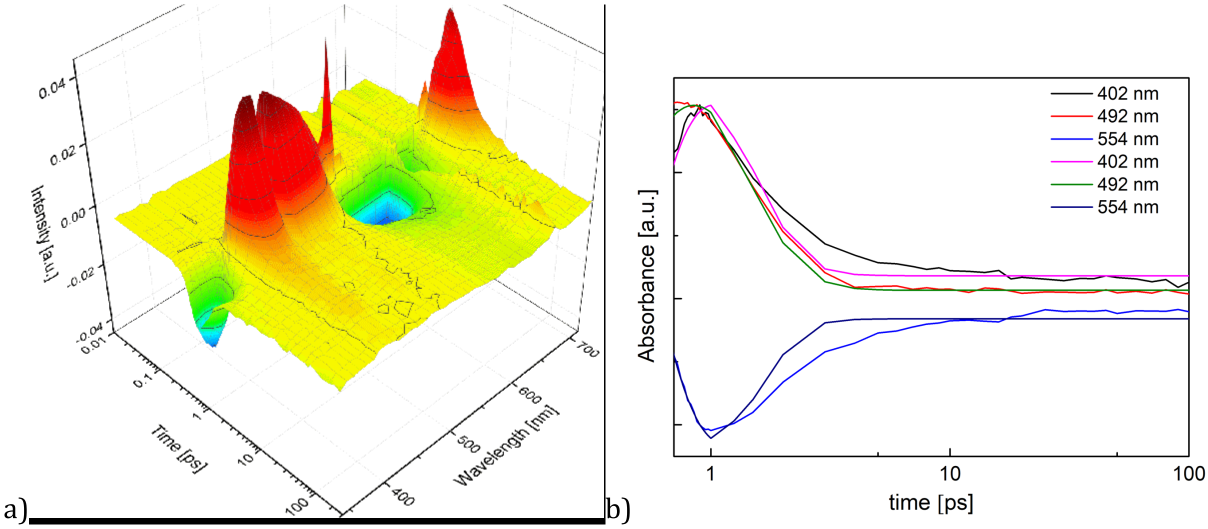

Figure S10: a) Spectra obtained of a solution of **1c** (non-degassed, CH_3CN, $4{\cdot}10^{-5}$ M, 25°C) upon irradiation with a 640 nm initial pulse in the femtosecond range until 300 ps; b) time profiles showing the decays of the absorbance at three different wavelengths. The fitted lines represent the modelled data as indicated in Table S1; the lack of fit was determined to be: 21.0 %. The large deviation originates most likely from the overlapping Raman-signals that were ignored in the fit.

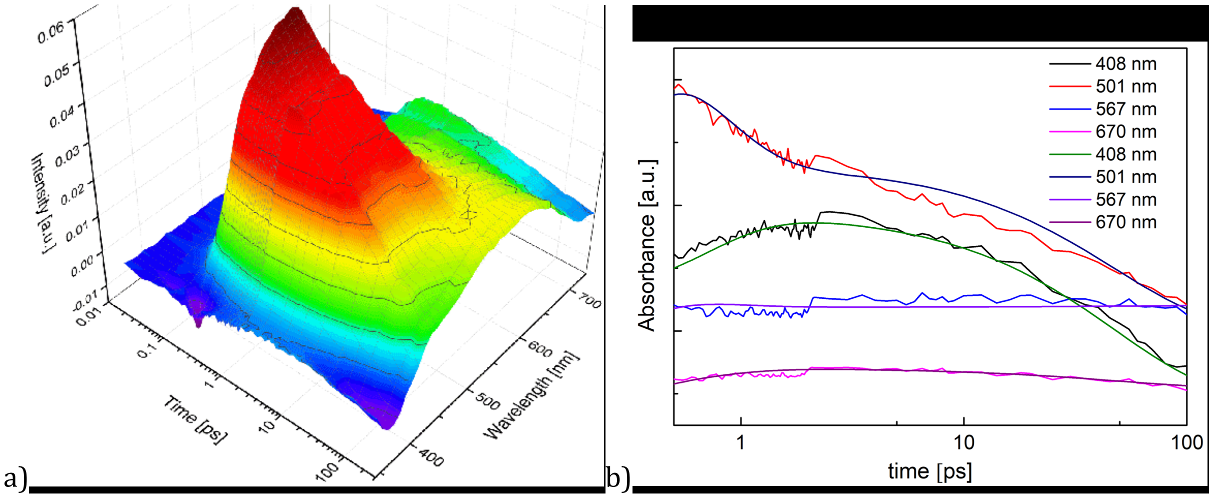

Figure S11: a) spectra obtained of a solution of **2o** (non-degassed, CH_3CN, $4{\cdot}10^{-5}$ M, 25°C) upon irradiation with a 320 nm initial pulse in the femtosecond range until 300 ps; b) time profiles showing the decays of the absorbance at three different wavelengths. The fitted lines represent the modelled data as indicated in Table S1; the lack of fit was determined to be: 5.8 %.

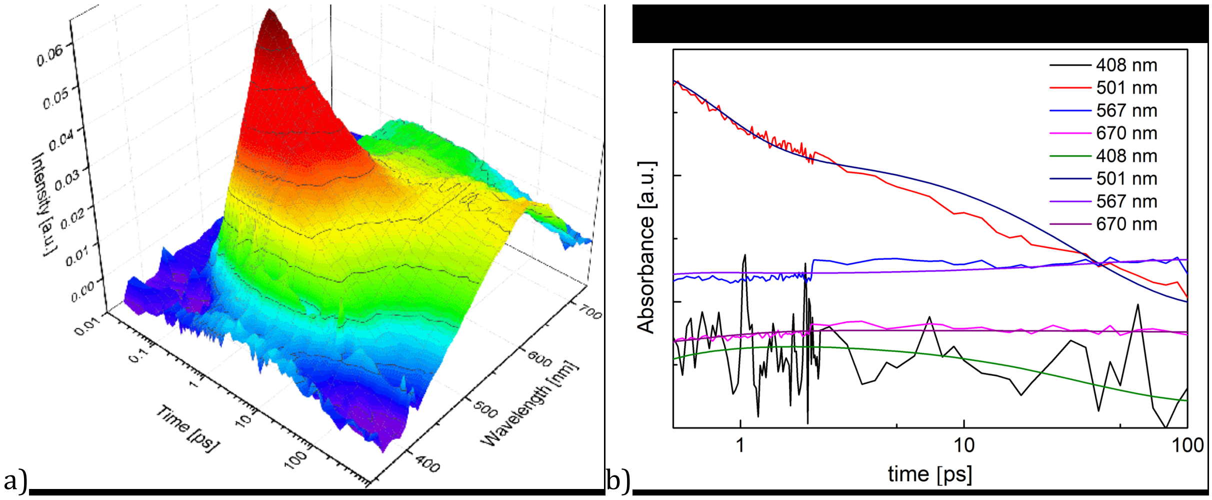

Figure S12: a) Spectra obtained of a solution of **2o** (non-degassed, CH_3CN, $4\cdot10^{-5}$ M, 25°C) upon irradiation with a 400 nm initial pulse in the femtosecond range until 1000 ps; b) time profiles showing the decays of the absorbance at three different wavelengths. The fitted lines represent the modelled data as indicated in Table S1; the lack of fit was determined to be: 6.1 %.

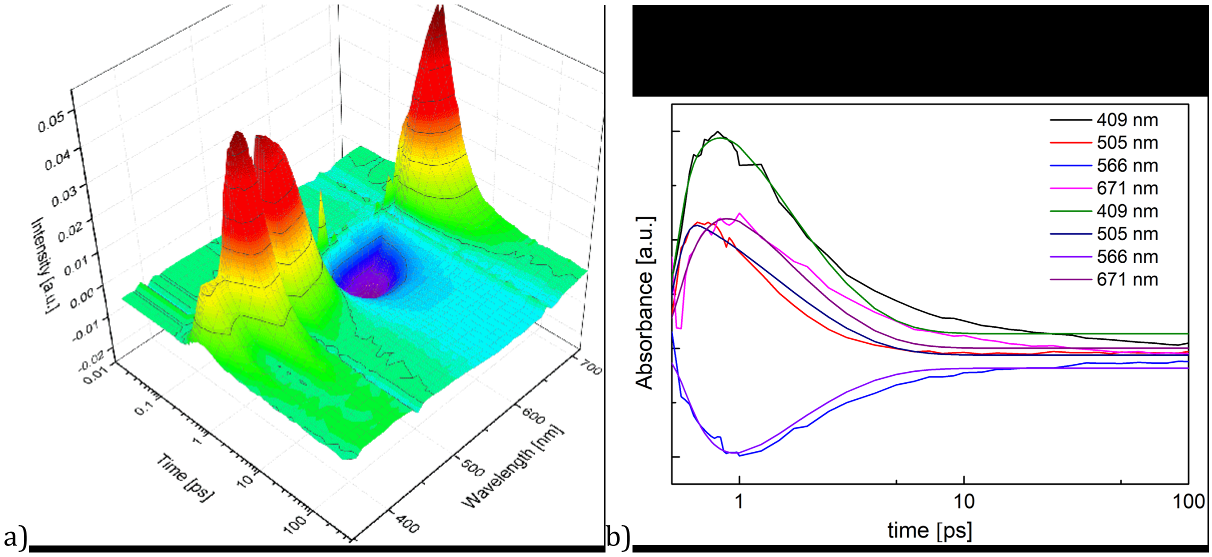

Figure S13: a) Spectra obtained of a solution of **2c** (non-degassed, CH_3CN, $4\cdot10^{-5}$ M, 25°C) upon irradiation with a 640 nm initial pulse in the femtosecond range until 500 ps; b) time profiles showing the decays of the absorbance at three different wavelengths. The fitted lines represent the modelled data as indicated in Table S1; the lack of fit was determined to be: 11.5 %.

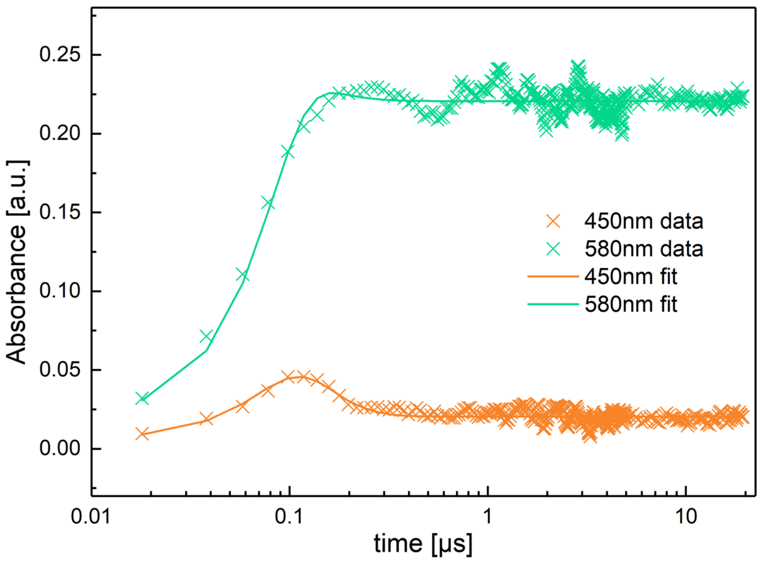

Figure S14: Decay traces at 450 nm (indicative of triplet state) and 580 nm (corresponding to the closed form) obtained for a solution of **2o** (non-degassed, CH_3CN, $4{\cdot}10^{-5}$ M, 25°C) upon irradiation with a 355 nm initial pulse in the nanosecond range until 20 µs.

Table S1: Summery of transient photochemical data of the investigated diarylethenes (absorbance ~ 1, CH_3CN, 25°C); *p* and *ap* indicate the parallel or antiparallel conformation.

DAE	proposed exc. state	decay τ	band maximum	active towards isomerization
**1o**	S_{FC}<br>$S_{relaxed}$<br>T	160 fs<br>5.9 ps<br>200 ns / 2.9 µs	570 nm<br>500 nm<br>670 nm	(no)<br>no<br>yes
**1c**	S_{FC}<br>$S_{relaxed}$	350 fs<br>700 fs	500 nm<br>430 nm	(no)<br>yes
**2o**	S_{FC}<br>$S_{relaxed}$<br>T (*p*)	650 fs<br>48 ps<br>60 ns / 3.8 µs	500 nm<br>430-500 nm<br>510 nm	(yes)<br>no<br>(yes)
**2c**	S_{FC}<br>$S_{relaxed}$	170 fs<br>1.5 ps	470 nm<br>430 nm	(no)<br>yes

3.4. Decay traces and Quenching experiments with carotene

In order to evaluate the spin state of the excited states of the long-living transient species observed in the nanosecond setup, quenching experiments were carried out using β-carotene. If the transient absorption belongs to a triplet state, it should be quenched by carotene so that the absorption of the investigated species decays much faster. Besides, the triplet excited state of β-carotene, which is not populated upon irradiation with λ_{exc} = 355 nm (generally applied wavelength in nanosecond setup), shows a high absorption at λ = 540 nm. An excitation energy transfer of the triplet excited state of the analyte toward carotene should therefore also be observable by new formed absorption at 540 nm.[8]

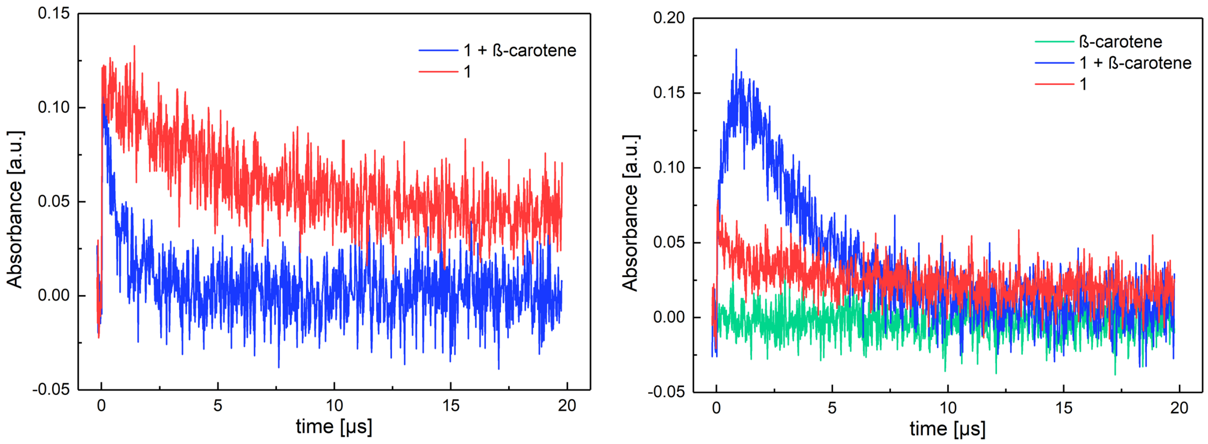

Figure S15: Decay traces of degassed solutions of **1**, β-carotene and a mixture of both upon excitation with a laser pulse of 355 nm. Left: observed at 600 nm indicative of the triplet excited state of **1**, efficiently quenched by carotene; Right: observed at 540 nm indicative of ^{3}β-carotene, efficiently populated via triplet-energy transfer from **1**.

[8] a) S. L. Bondarev and S. M. Bachilo, *J. Appl. Spectrosc.* **1994**, *60*, 229-233; b) S. M. Bachilo, *J. Photoch. Photobio. A* **1995**, *91*, 111-115.

3.5. Excitation Schemes

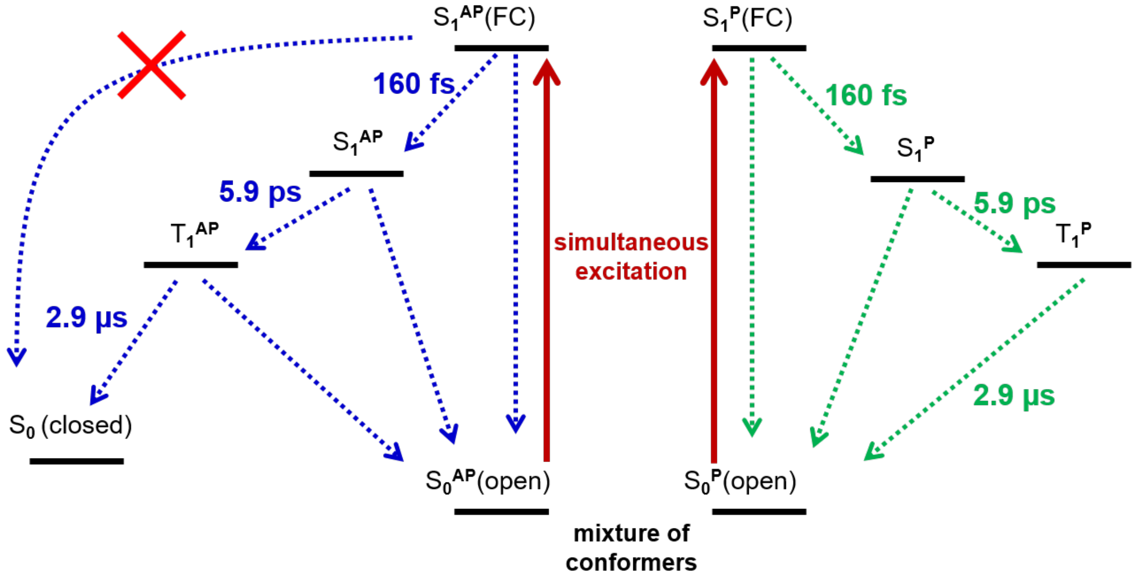

Figure S16: Full excitation scheme for the irradiation of **1o** with 400 nm and 355 nm light.

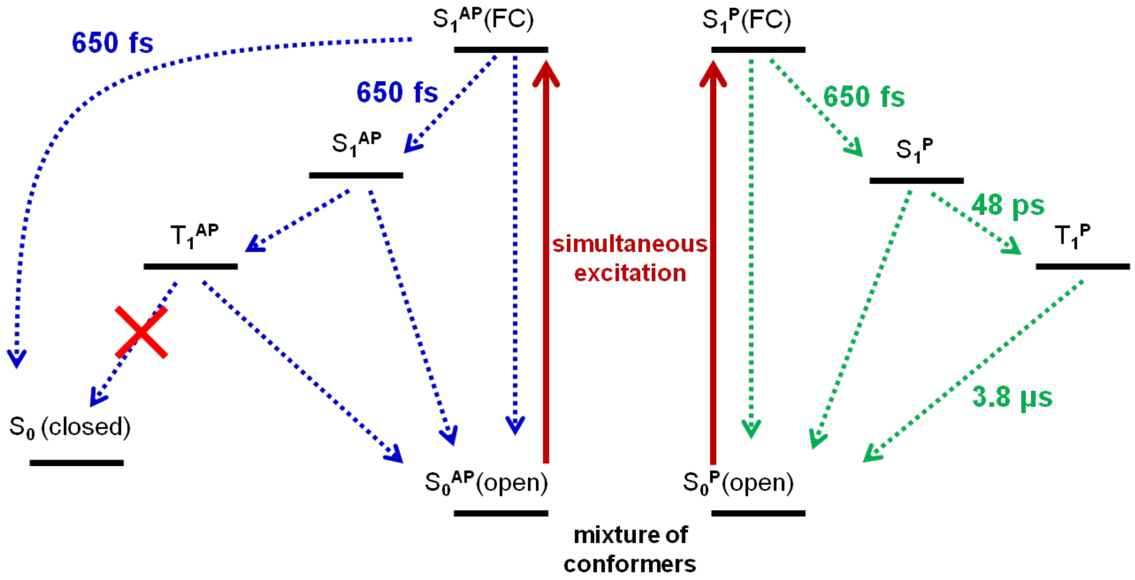

Figure S17: Full excitation scheme for the irradiation of **2o** with 400 nm and 355 nm light.

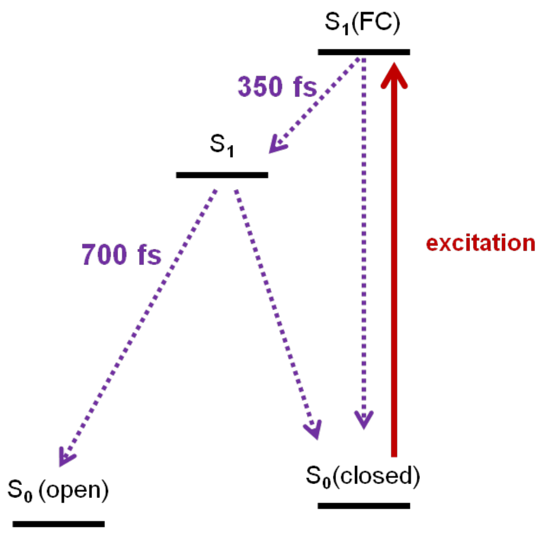

Figure S18: Full excitation scheme for the irradiation of **1c** with 640 nm light.

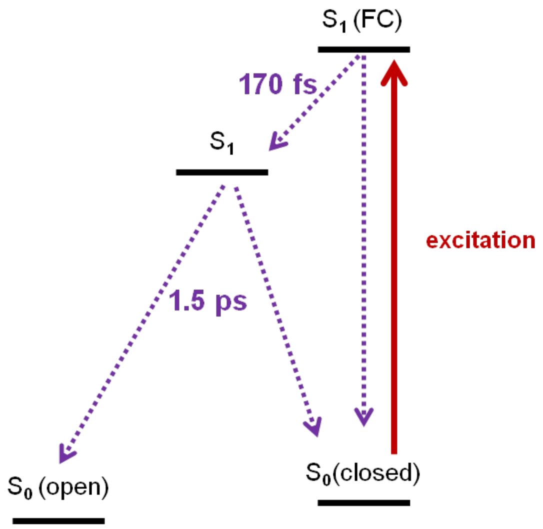

Figure S19: Full excitation scheme for the irradiation of **2c** with 640 nm and 355 nm light.

3.6. Fluorescence of 2c

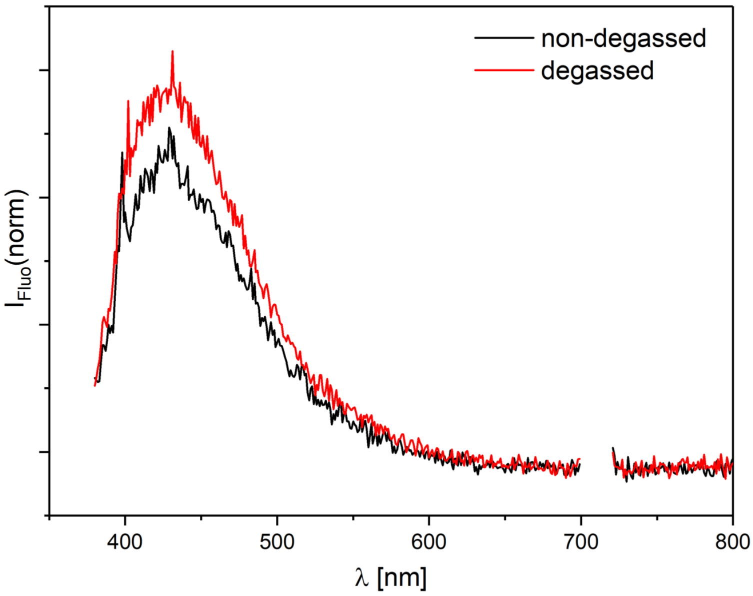

Figure S20: Fluorescence of **2c** normalized to absorbance at irradiation wavelength λ_{irr} = 355 nm (CH_3CN, 25°C, ~$1 \cdot 10^{-5}$ M); no phosphorescence was observed under these conditions.

4. NMR-spectra of 2

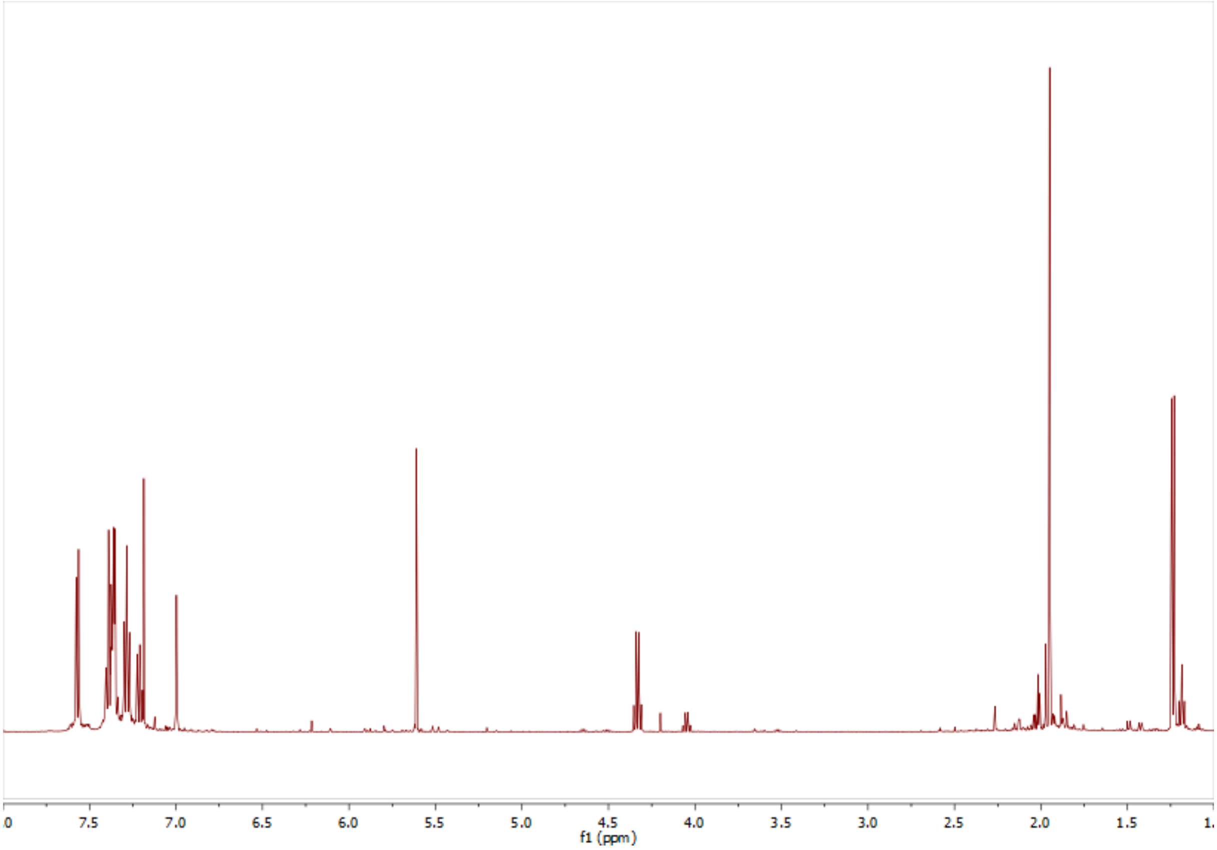

Figure S21: ^{1}H-NMR-spectrum of compound **2o**.

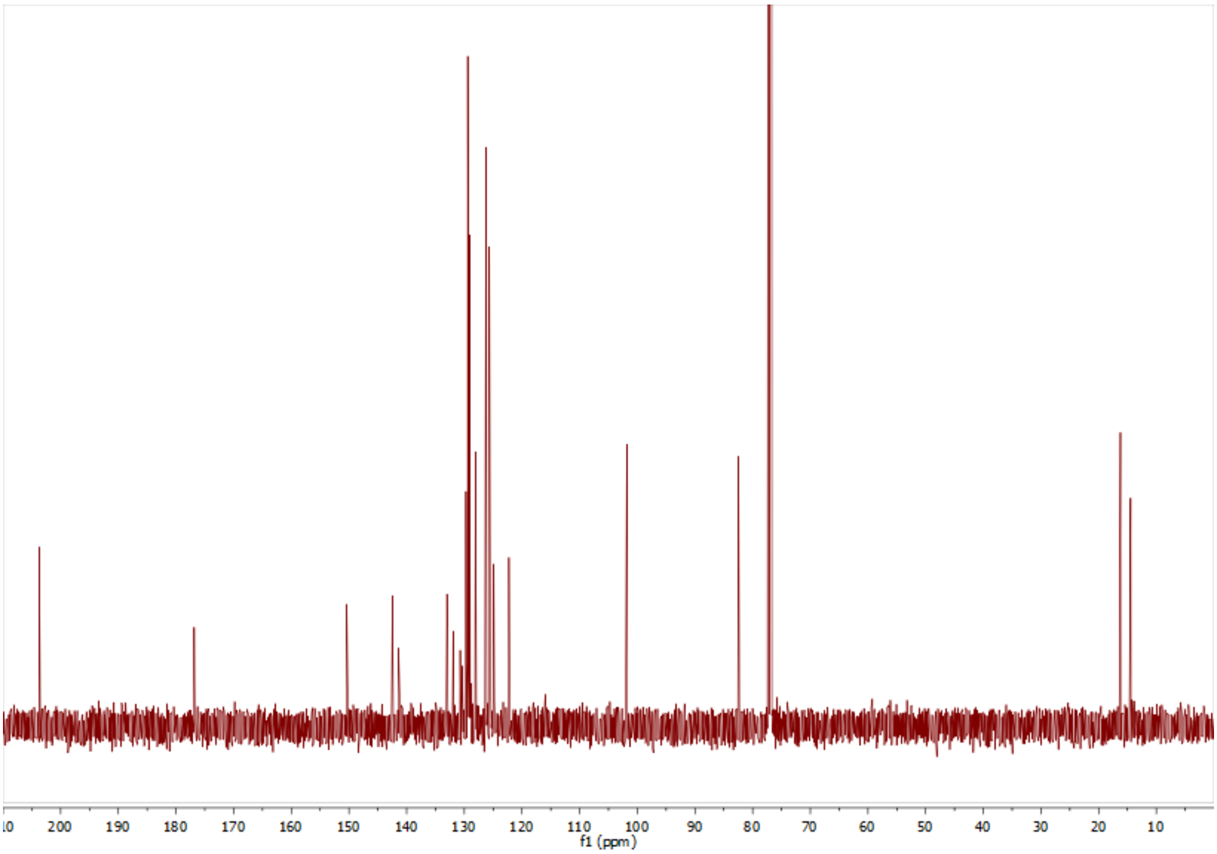

Figure S22: ^{13}C-NMR-spectrum of compound **2o**.

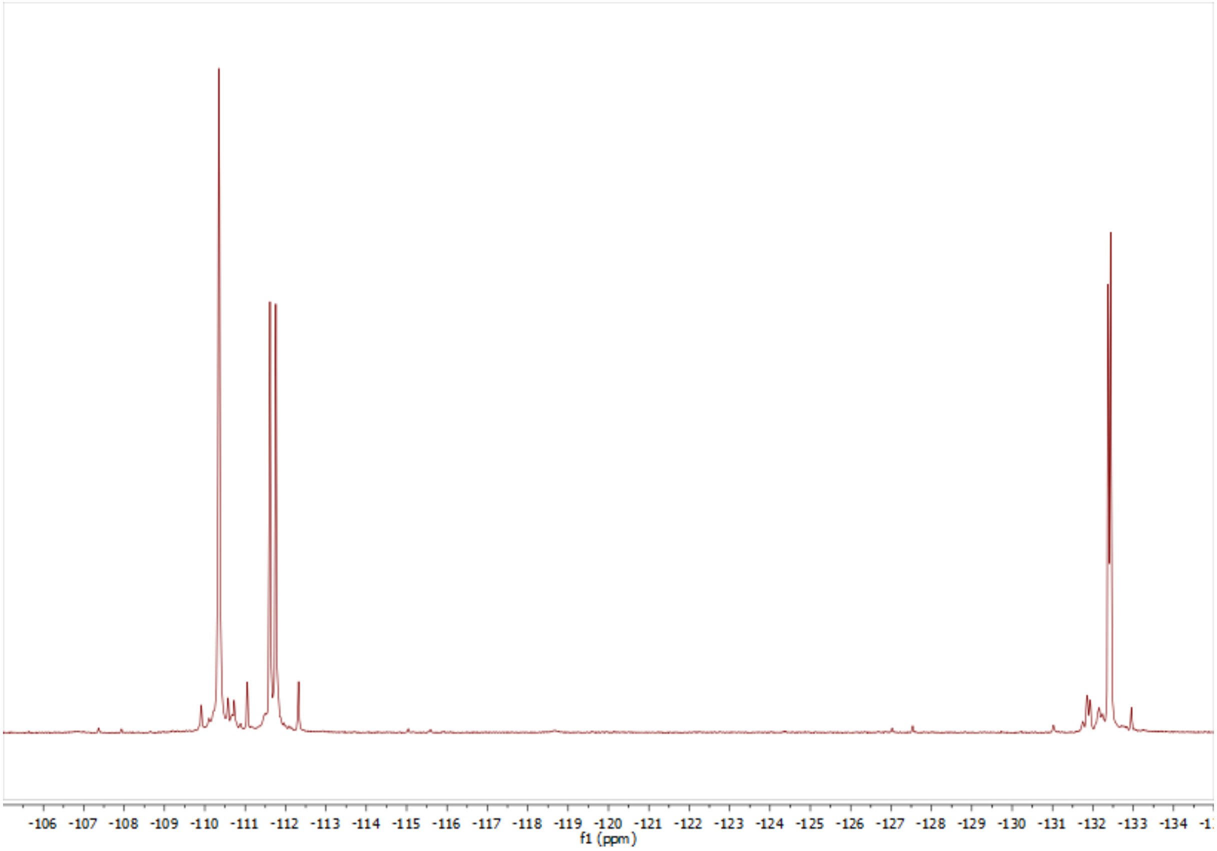

Figure S23: ^{19}F-NMR-spectrum of compound **2o**.
